# Comparison of efficiency and safety of open surgery, hybrid surgery and endovascular repair for the treatment of thoracoabdominal aneurysms: a systemic review and network meta-analysis

**DOI:** 10.3389/fcvm.2023.1257628

**Published:** 2023-12-15

**Authors:** Tinghua Liu, Jiani Zhao, Jinjian Sun, Kemin Wu, Wei Wang

**Affiliations:** ^1^ Department of Vascular Surgery, Xiangya Hospital, Central South University, Changsha, China; ^2^ National Clinical Research Center for Geriatric Disorders, Xiangya Hospital, Central South University, Changsha, China

**Keywords:** thoracoabdominal aorta aneurysm, open surgical repair, endovascular, hybrid surgery repair, network meta-analysis (NMA)

## Abstract

**Objective:**

The objective of this study was to perform a network meta-analysis (NMA) to assess the efficacy and safety of three different surgical interventions- open surgical repair (OSR), hybrid surgical repair (HSR), and endovascular repair (EVAR)- for the treatment of thoracoabdominal aortic aneurysms (TAAAs).

**Methods:**

Electronic repositories like PubMed, Embase, Web of Science, Scopus, ScienceDirect, the Cochrane library, Clinical trial, and China National Knowledge Infrastructure (CNKI) were systematically searched to identify studies that compared the efficacy of OSR, HSR, and EVAR with endografts for the treatment of TAAAs until December 24th, 2022. Random-effects and fixed-effects models were employed to analyze the data gathered in a network meta-analysis. The study's primary outcomes of interest encompassed in-hospital mortality, long-term survival rate, and postoperative complications.

**Results:**

Eleven comparative studies meet inclusion criterias. There were 2,222 patients in OSR, 1,574 patients in EVAR and 537 patients in HSR. EVAR has lower one-month mortality than OSR (RR: 0.31; 95% CI: 0.17–0.70) and HSR (RR: 0.37; 95% CI: 0.22–0.71), and lower incident rate of renal complications than HSR (RR: 0.20; 95% CI: 0.08–0.43) and OSR (RR: 0.34; 95% CI: 0.16–0.65). Nonetheless, there was no noteworthy discrepancy identified in the long-term survival rates of these procedures.

**Conclusions:**

As compared with OSR, HSR, and EVAR, EVER has lower one-month mortality, and lower incident rates of complications.

**Systematic review registration:**

PROSPERO (CRD42022313829).

## Introduction

In relation to aortic aneurysms, thoracoabdominal aneurysms (TAAAs), which make up 10% of all aortic aneurysms in the body, are life-threatening diseases with high mortality and a high incidence of complications (1, 2). According to statistics, when the diameter of the TAAAs reaches 7 cm, it has a >40% chance of undergoing rupture without treatment. The two-year fatality rate is 76%, and the five-year fatality rate is more than 95% (3, 4). TAAAs has a wide range of lesions and a poor natural prognosis, and it especially involves multiple visceral arteries, which makes it difficult to treat. At present, for TAAAs, there is no effective conservative treatment available. In order to treat TAAAs, three major treatments are available: open surgical repair (OSR), hybrid surgery repair (HSR), and endovascular repair (EVAR) (6).

E. Stanley Crawford performed the first successful OSR, aimed at protecting organs and preventing recurrent aneurysms, and the OSR was refined by Crawford et al., who reported lighter surgical trauma and higher surgical safety in 1978 (7). For over six decades, it was the gold standard for treating TAAAs. Modern open techniques include heparinization, mild permissive hypothermia, intercostal artery reimplantation, cold renal perfusion, cerebral spinal fluid (CSF) drainage, selective visceral perfusion, and left heart bypass (LHB) for extensive TAAA repairs (namely Crawford extent I and II TAAA repairs) (8).

The hybrid procedure, which Quinones-Baldrich and colleagues introduced in 1999, involves one or two stages (separated by days, weeks, or months) (9). Prior to endovascular exclusion of the TAAA, a carotid to subclavian bypass or retrograde debranching of the common iliac arteries is performed. The procedure entails redirecting visceral and renal arteries- including the celiac axis, superior mesenteric, left renal, and right renal arteries- using bypass grafts that are roughly 8–10 mm in diameter, followed by aortic reattachment either below or above the endovascular zone. An endovascular exclusion of the aneurysm covers the vessel origins once the repair has been completed (10).

A less invasive option, reinforced fenestrations or directional branches, for TAAA repair via endovascular means was introduced in the late 1990s and early 2000s as a substitute for open surgery (11). The technique has evolved from using physician-modified endovascular grafts (PMEGs) to patient-specific and off-the-shelf devices. Aortic centers have perfected technique and perioperative care while improving clinical outcomes along with several improvements in device design. Complex aortic aneurysms are commonly treated with fenestrated-branched endovascular aortic repair (FB-EVAR) (12).

The use of large-centre EVAR can match any open surgery repair with a low early mortality, but long-term follow-up clinical trials are lacking, we do not yet know the long-term survival effects of EVAR (13, 14). According to early reports, HSR has lower perioperative complication rates than open repair, but there are no prospective comparisons (15). What's more, different articles are controversial concerning the short-term mortality between OSR and HSR. All of these techniques have advantages and disadvantages. To offer a reference point for the management of the condition, the study evaluated the effectiveness and safety of these approaches in treating TAAAs.

## Materials and methods

This systematic review and network meta-analysis of TAAA treatments has been registered under the number *CRD42022313829* with PROSPERO- a branch of the National Institute for Health Research, in alignment with the PRISMA NMA guidelines (16) (Supplementary Table S1).

### Search strategy

We searched the following databases for included studies: Scopus, Embase, the Cochrane library, and PubMed, ScienceDirect, Clinical trial, Web of Science, and China National Knowledge Infrastructure (CNKI), and we ensured that the studies were published before December 24th, 2022. The main search words were “thoracoabdominal aortic aneurysm”, “open surgical repair”, “endovascular repair”, and “hybrid repair”. The search strategy is shown in Supplementary Table S2. Furthermore, no language restriction or eligible articles were excluded.

### Selection criteria

Study inclusion criteria were as follows:

(1) P (patients): patients presenting with TAAAs;
(2) I (Intervention) and C (comparison): OSR vs. HSR vs. EVAR;
(3) (outcomes): primary outcomes: including efficiency (1-month mortality, 6-month, 1-, 3-, and 5-year long-term survival rate); secondary outcomes: including complications (cardiac disease, pulmonary complications, renal complications spinal cord ischaemia, and stroke);
(4) S (studies): the selection parameters included randomized controlled trials (RCTs) or cohort studies (S-studies).

The exclusion criteria were: (1) articles with duplicated data or solely abstracts; (2) conference articles, animal studies, or meta-analyses; (3) articles unrelated to TAAAs; and (4) articles without original data.

### Data extraction

The study was conducted by two independent investigators who extracted the article title, first author, publication year, type of study, follow-up duration, nation, participant count, baseline data on participants (such as age, sex, BMI, aortic diameter, Crawford classification, and information on other underlying diseases), average procedure time, average blood loss, in-hospital mortality, and incidence of complications, including stroke, paraplegia/spinal cord ischemia, cardiac issues, pulmonary issues, and renal issues. Both emergency and non-emergency surgical cases were included. To obtain a comprehensive understanding of the long-term effectiveness of these procedures, we assessed the survival rates at 3 months, 6 months, 1 year, 2 years, and 3 years. A third investigator was involved in the assessment process in the event of divergent opinions.

### Statistical analysis

A random effects NMA was conducted using the BUGSnet package, operated through R Studio (R Foundation for Statistical Computing, Vienna, Austria) (17). BUGSnet produced relative risks (RRs) with 95% confidence intervals for dichotomous event data. For ranking interventions, the sum under the cumulative ranking (SUCRA) score was used. SUCRA is a measure that indicates whether an intervention is among the best options, expressed as a percentage.

We utilized the Newcastle-Ottawa Scale (NOS) to assess the quality of cohort studies based on comparability, exposure, and selection. The scale consists of nine points, and studies with an overall score between 8 and 9 were evaluated as high-quality, while those with 6–7 points were medium-quality (18). Additionally, we employed the Grading of Recommendations Assessment, Development, and Evaluation (GRADE) system to determine the level of evidence, which included evaluating items like inconsistency, bias risk, imprecision, indirectness, and publication bias (Supplementary Figures S5–S11). Based on this methodology, evidence was classified as high, moderate, low, or very low (19).

To evaluate the pairwise meta-analysis, Review Manager 5.3 (Nordic Cochrane Centre, Oxford, UK) was utilized, where heterogeneity was assessed using the *I^2^* statistic and *χ*^2^ test. A fixed-effects model was employed when no significant heterogeneity was detected (*I^2^* < 50% or *p* > 0.1), while a random-effects model was used otherwise. For dichotomous variables such as mortality, long-term survival rate, and complications, RRs with 95% CIs were employed. Publication bias and contribution plots were generated with STATA 12.0 (Stata Corp, Texas, USA), while outcomes between different treatments were compared using the BUGSnet package in R Studio (R Foundation for Statistical Computing, Vienna, Austria).

## Results

### Search results and study characteristics

**Figure 1:**
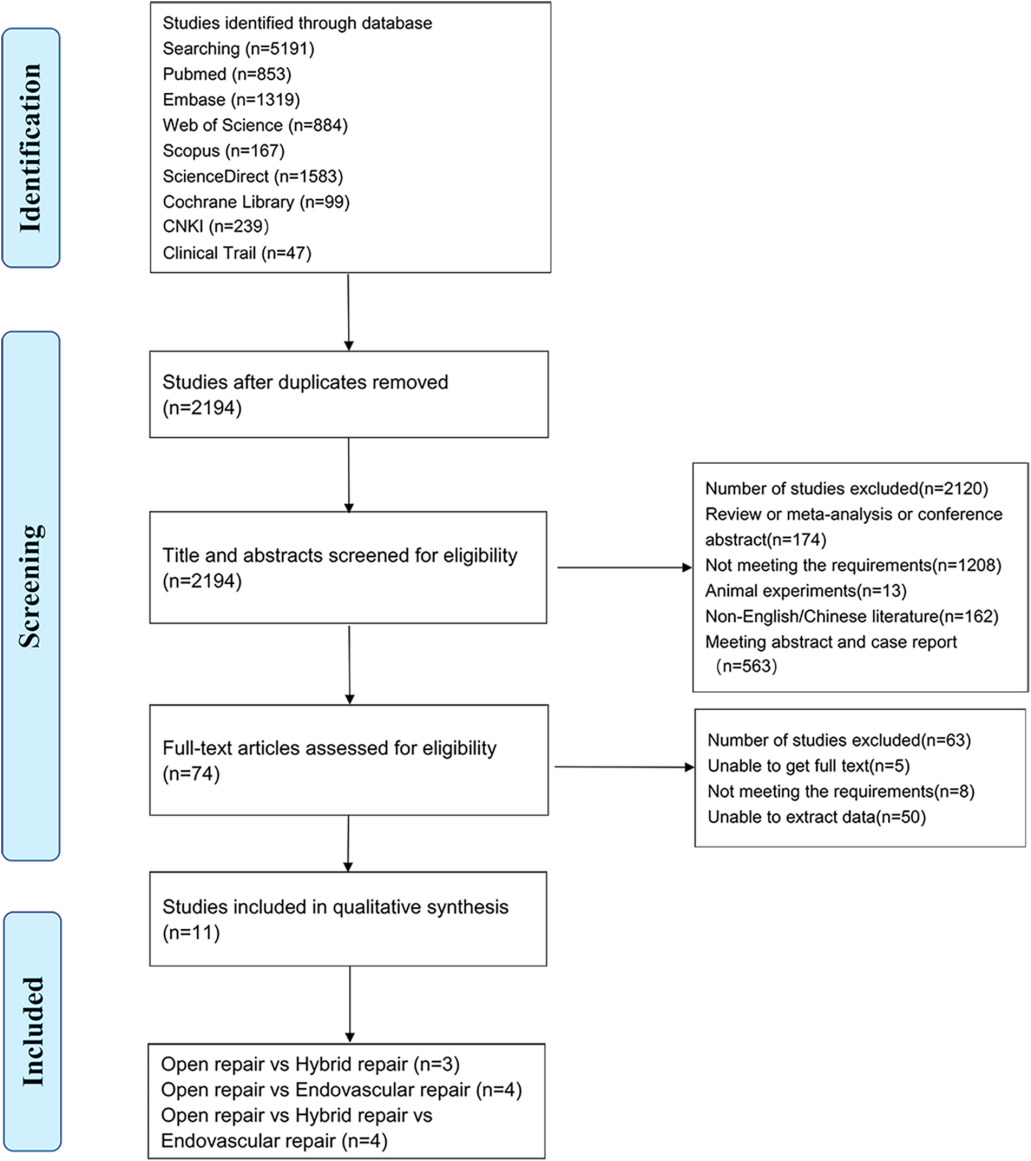
Preferred reporting items for systematic reviews and meta-analysis (PRISMA) flow diagram of identification, screening, eligibility, and inclusion phases of the systematic search for studies providing comparative outcomes between methods of thoracoabdominal aortic aneurysms (TAAA) repair.

Included in the study were 11 articles involving a total of 4,333 patients (5, 20–29), with three groups identified: OSR group (2,222 patients), EVAR group (1,574 patients), and HSR group (537 patients). The detailed search process is illustrated in Figure 1, with the included studies ranging in publication from 2007 to 2019 and the median age of patients ranging from 45 to 76 years (5, 20–29). Baseline characteristics of the studies are presented in Table 1, and were found to be comparable across all groups. As shown in Supplementary Table S3, the quality of the studies is presented, while the level of evidence of the results is presented in Supplementary Table S4.

**Table 1:** Characteristics of included studies.

Study	Country	Groups	Patients (*n*)	Male/female	Median age (year)	Follow up time (month)	Maximum aortic dimension (cm)	Crawford				Surgical indication		Operation duration (min)	Bleeding volume (ml)	Previous aortic surgery	Re-intervention (%)	Technical success (%)
								Type I	Type II	Type III	Type IV	Emergent (*n*)	Elective (*n*)					
Kang (5)	America	OSR	54	36/18	62	3.9—38.1	6.8	4	18	10	22	—	—	—	—	—	—	—
		HSR	24	15/9	63.8	3.9—38.1	6.8	2	16	2	4	—	—	—	—	—	—	—
		EVAR	68	51/17	74.9	3.9—38.1	6.7	10	15	14	29	—	—	—	—	21	—	—
Chiesa (20)	French	OSR	25	25/0	63.5	11day—57.4	—	10	5	4	7	—	—	169	1,350	—	—	100
		HSR	13	12/1	69.6	—	—	7	2	0	2	1	—	239	480	—	—	100
Patel (21)	America	OSR	73	44/29	59.5	4.5—56.5	6.6	18	38	17	—	6	53	227.4	—	26	—	—
		HSR	29	7/22	71.7	4.5—56.5	6.3	1	12	16	—	4	23	—	—	17	—	100
Ci (22)	China	OSR	9	5/4	51	3—12	—	—	—	—	9	—	—	—	800	—	—	100
		HSR	1	1/0	45	3	—	—	—	1	—	—	—	—	800	—	—	100
Benrashid (23)	America	OSR	84	57/27	66	1—60	6.3	20	11	24	29	14	70	173	—	41	1.2	—
		HSR	81	41/40	72	1—60	6.4	7	34	37	0	15	66	—	—	48	12.3	97.5
Ferrer (24)	Italy	OSR	65	49/16	70.7	1—48	—	8	16	17	24	12	53	—	—	21	12.3	—
		EVAR	65	51/14	70.7	1—48	—	6	17	20	22	1	64	—	—	30	15.4	—
Feng (25)	China	OSR	8	6/2	53.6	2—72	—	—	—	—	—	—	—	480	778.6	—	—	—
		HSR	3	3/0	50.7	2—72	—	—	—	—	—	—	—	462	733.3	—	—	—
		EVAR	11	9/2	53.4	2—72	—	—	—	—	—	—	—	240	205.6	—	—	—
Locham (26)	America	OSR	398	234/164	66.5	1	—	—	—	—	—	107	290	—	—	—	—	—
		EVAR	481	251/230	71.2	1	—	—	—	—	—	121	354	—	—	—	—	—
Geisbüsch (27)	Germany	OSR	1,422	939/483	67	1	—	—	—	—	—	—	—	—	—	—	—	—
		HSR	346	228/118	67	1	—	—	—	—	—	—	—	—	—	—	—	—
		EVAR	839	589/250	67	1	—	—	—	—	—	—	—	—	—	—	—	—
Bertoglio (28)	Italy	OSR	18	14/4	76	1	—	—	—	—	—	—	—	—	—	—	—	—
		EVAR	18	15/3	70	1	—	—	—	—	—	—	—	—	—	—	—	—
Arnaoutakis (29)	America	OSR	66	48/18	59	1—60	6.5	—	26	40	—	–	—	—	—	11	—	—
		HSR	40	27/13	71	1—60	6.7	—	12	28	—	—	—	—	—	30	—	—
		EVAR	92	57/35	72	1—60	6.6	—	5	48	—	—	—	—	—	—	—	—

### Publication bias

To evaluate the risk of bias, the NOS system for cohort studies was employed. Out of the 11 studies, 3 were deemed to have a high risk of bias, and 8 were assigned a moderate risk of bias. It was found that there was inadequate compensation for selection bias due to differences in physiological fitness and Crawford types among the treatment arms for OSR, HSR, and EVAR, as seen in the Supplementary Material across various studies.

### Mortality

**Figure 2:**
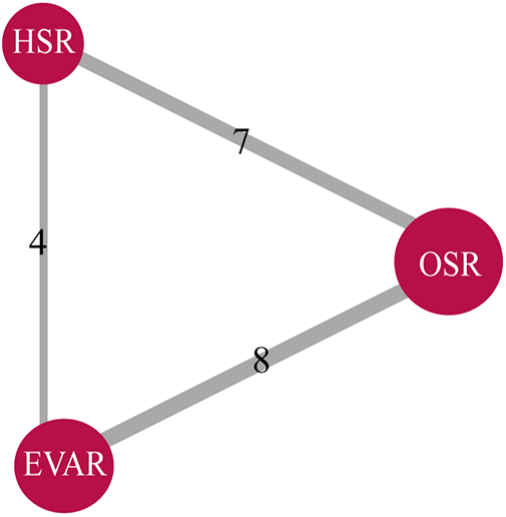
Literature summary network plots for all-cause 1-month mortality (4,333 patients across 11 studies) in studies providing comparative outcomes between methods of thoracoabdominal aortic aneurysms (TAAA) repair. The size of each red node corresponds to the number of study arms included for a treatment across all comparisons. The width of each grey line corresponds to the number of studies comparing the two interventions directly. OSR, open surgical repair; HSR, hybrid surgery repair; EVAR, endovascular repair.

For the purpose of evaluating 1-month mortality across the three techniques, 11 articles were included in this NMA, as illustrated in Figure 2. In this network, a total of 4,333 patients were included, and 725 deaths (16.7%) were reported as a result.

**Figure 3:**
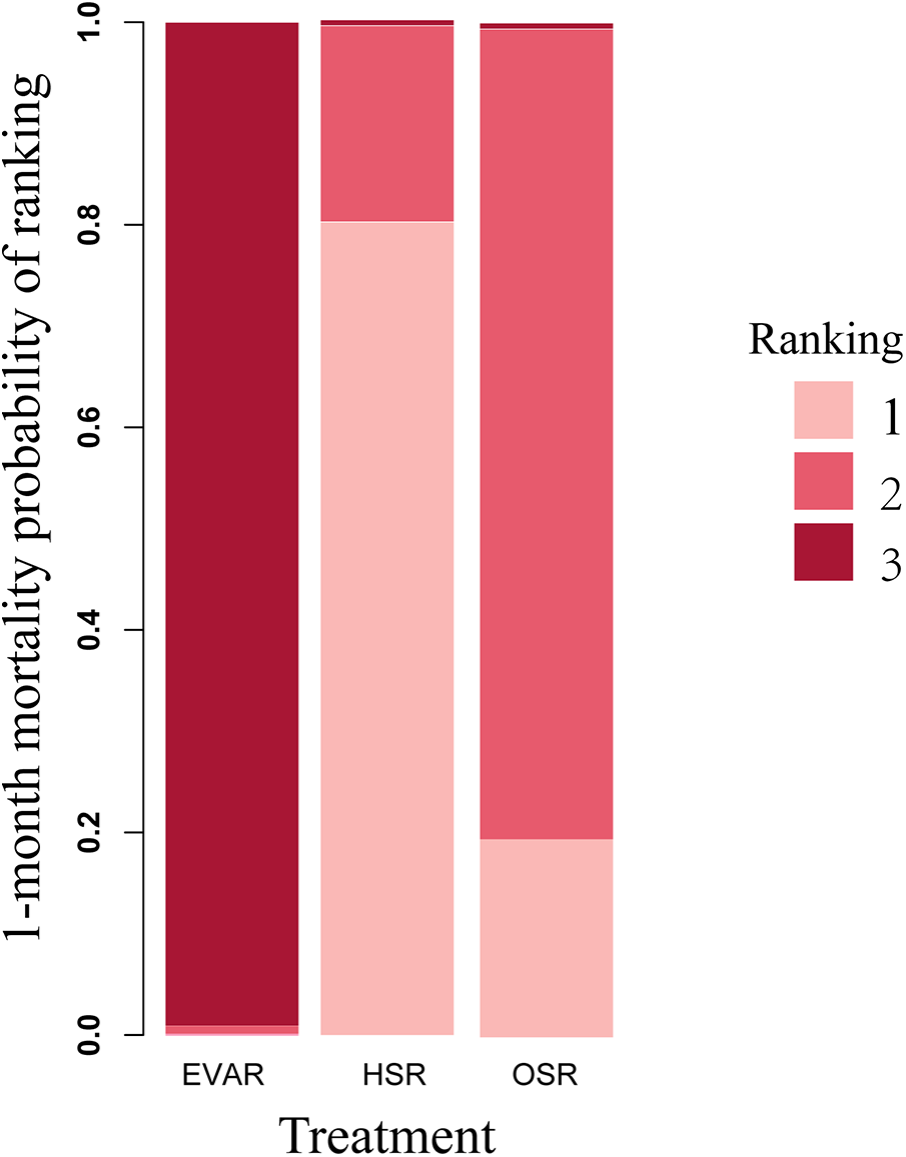
Rank probability for all-cause mortality network meta-analysis 1-month mortality (4,333 patients across 11 studies; open surgery 2,222 patients, HSR 537 patients, and EVAR 1,574 patients), displaying the probability that each treatment is the best treatment, where higher rankings are associated with smaller outcome values. OSR, open surgical repair; HSR, hybrid surgery repair; EVAR, endovascular repair.

According to the head-to-head comparison illustrated in Supplementary Figure S1, EVAR exhibited a lower 1-month mortality rate compared to both HSR (RR: 0.31; 95% CI: 0.17–0.70) and OSR (RR: 0.37; 95% CI: 0.22–0.71). However, no significant difference was observed between the HSR and OSR groups (RR: 1.22; 95% CI: 0.66–1.98). The rank probability indicated that EVAR had the lowest 1-month mortality while HSR had the highest 1-month mortality, as displayed in Figure 3.

### Long-term survival rate

**Figure 4:**
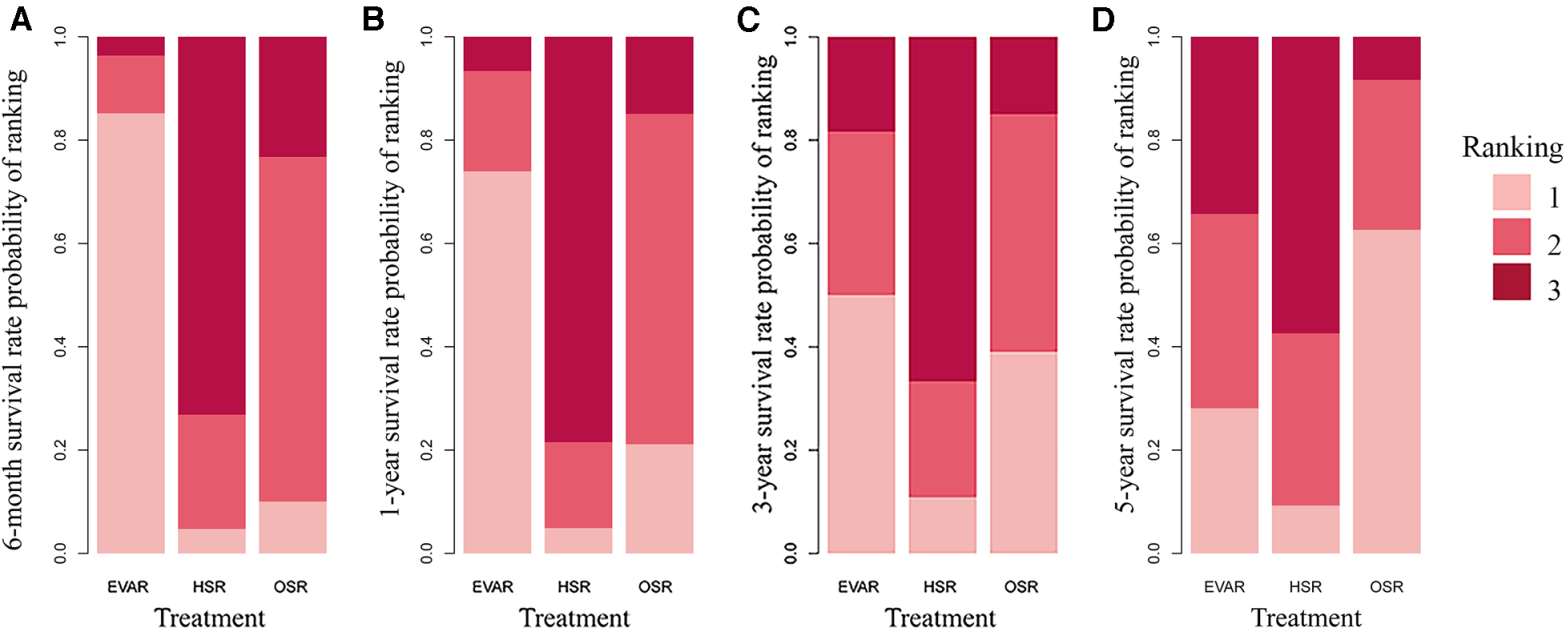
Rank probability for comparative survival rate network meta-analysis at 6-month (A), 1-year (B), 3-year (C) and 5-year (D) rate (4,333 patients across 11 studies; open surgery 2,222 patients, HSR 537 patients, and EVAR 1,574 patients), displaying the probability that each treatment is the best treatment, where higher rankings are associated with smaller outcome values. OSR, open surgical repair; HSR, hybrid surgery repair; EVAR, endovascular repair.

In our analysis, we evaluated the long-term survival rates of these techniques at 6 months, 1 year, 3 years, and 5 years. A total of eight articles were used for this analysis, with Supplementary Figure S2 providing additional details. Following a head-to-head comparison, we found no statistically significant differences in survival rates among the OSR, EVAR, and HSR groups for the aforementioned time periods (Supplementary Figure S3). Additionally, the rank probability suggested that EVAR may be a favorable option for TAAA treatment in terms of a lower 1-month survival rate and higher overall survival rate, as illustrated in Figure 4.

### Complications

The major complications of these procedures included cardiac disease, pulmonary complications, renal complications, spinal cord ischaemia, and stroke.

Renal complications were found to be the most frequently occurring complication across all three treatment groups (Table 2). According to our analysis illustrated in Supplementary Figure S4, EVAR was associated with a lower incidence of renal complications compared to HSR (RR: 0.20; 95% CI: 0.08–0.43) and OSR (RR: 0.34; 95% CI: 0.16–0.65). Additionally, comparing EVAR to OSR, EVAR had a lower incidence of pulmonary complications (RR: 0.13; 95% CI: 0.02–0.47).

**Table 2:** The Top 5 complications associated with OSR, HSR and EVAR.

Group	Complication	Study	Event/ total	Frequency (%)
OSR	Renal complications	11	950/1,812	52.43
	Pulmonary complications	8	150/737	20.35
	Cerebrovascular disease	4	203/1,911	10.62
	Spinal Cord ischemia	9	144/2,188	6.58
	Stroke	5	43/2,043	2.10
HSR	Renal complications	7	319/566	56.36
	Pulmonary complications	5	27/166	16.27
	Spinal cord ischemia	6	26/523	4.97
	Cerebrovascular disease	3	13/399	3.26
	Stroke	4	9/496	1.81
EVAR	Renal complications	7	497/1,574	31.58
	Pulmonary complications	5	55/667	8.25
	Cerebrovascular disease	3	94/1,412	6.66
	Spinal cord ischemia	7	94/1,574	5.97
	Stroke	3	16/1,412	1.13

## Discussion

TAAAs are a disease with a high mortality; however, the management of TAAAs remains formidable. Our results showed that, compared with the other two procedures, EVAR has lower 1-month mortality and fewer complications.

Initially, a head-to-head comparison was conducted to assess the efficiency of the three procedures. The outcome suggested that EVAR had a lower 1-month mortality rate when compared to HSR and OSR groups. Although EVAR had lower incidence rates for renal and pulmonary complications, long-term survival rates were not significantly different among the three techniques. The following are the reasons behind the lower 1-month mortality rates associated with EVAR: (1) By performing these procedures percutaneously, the need for extensive surgical techniques such as thoracolaparotomy, aortic cross clamping, and cardiopulmonary bypass is eliminated. (2) The continuous flow provided to the renal-mesenteric vessels and lower extremities significantly reduces the hemodynamic impact, blood loss, physiological stress and risk of end-organ ischemia (30). During EVAR procedure, there will be no sudden massive bleeding, sharp fluctuations in blood pressure and heart rate, the patient's vital signs will be relatively stable. Furthermore, patients choose hybrid surgery, often due to complications or other high-risk patients with open surgery, or TAAA rupture and perfusion, and cases requiring emergency surgery with poor injection. Ferrer et al. reported that the EVAR group had lower mortality than the HSR and OSR groups (23). Additionally, Arnaoutakis et al. found no significant difference between the three groups (28). The study has a small number of patients, this may lead to such results. We noticed that our result of the 1-month mortality rate of HSR (26.0%) was higher than the others (14.3%) (31). Geisbüsch's study (25), which included 2,607 people, showed that the 1-month mortality risk of HSR is as high as 30.9%, which has a greater impact on the overall outcome. The authors of this study stated that in low-level hospitals, hybrid surgery for thoracoabdominal aortic aneurysm had a higher mortality rate, and this study also included more data from low-level hospitals, which affected the final results. Moreover, the patients in the HSR group had more basic diseases than those in the OSR group; therefore, it also had an effect on the 1-month mortality rate. Similar findings were observed in the open surgery group, with OSR still showing a high mortality rate in most centers. After three decades of experience and treating over 3,500 patients, a significant milestone was achieved with the lowest mortality rate recorded at 7.5%. It is worth noting that the highest survival rate was observed in the largest series ever reported, suggesting that treatment outcomes can be improved at high-volume centers (30).

To confirm the long-term efficiency among these procedures, we determined the survival rates for 6 months, 1 year, 3 years, and 5 years of these techniques. EVAR had longer survival rates at 1 year than OSR. In addition, HSR had a higher 3-year survival rate than OSR. This result may indicate that EVAR may have a lower 1-month mortality and a higher early survival rate than the other groups. EVAR has obvious minimally invasive advantages, but the operation requires vascular reconstruction, the highly specialized skill required for this makes it extremely challenging to apply universally, as it heavily relies on the surgeon or center, so emergency surgery is generally not recommended. Moreover, EVAR had the lowest 30-day mortality and higher long-term survival, although contrast medium was used during surgery. However, if the perioperative period can be safely managed, the efficiency of OSR is positive. Furthermore, long-term survival was not different between OSR and EVAR. Compared with EVAR, the long-term reintervention rate of OSR is significantly lower. Due to the fact that younger patients have longer lifespans, OSR can be recommended for young patients, and EVAR is beneficial to older patients.

To evaluate the safety of the three procedures, we compared the incidence of complications after the treatments. Cardiac disease, pulmonary complications, renal complications, spinal cord ischaemia, and stroke. Based on our results, EVAR had a lower incidence rate of pulmonary complications than OSR. OSR requires incision of sternum and suprarenal or supraceliac clamping, which creates further stress and ischemia-reperfusion injury to lungs and intraabdominal viscera. Research has shown that patients who receive endovascular repair tend to be older and have a higher incidence of comorbidities such as coronary artery disease, chronic obstructive pulmonary disease (COPD), and chronic kidney disease. However, these patients have a lower incidence rate of complications compared to the other two groups, which is consistent with our findings. Feng et al. showed that EVAR had the lowest perioperative 30-day complication rate, with rates of 9.1% (1/11), 62.5% (5/8), and 66.7% (2/3) in the OSR, HSR, and EVAR groups, respectively, and it may become the first choice for treating TAAA. Verhoeven et al. and Sultan et al. found that EVAR had lower mortality and complication rates. For OSR, to a large extent, the prognosis of traditional open surgery for thoracic and abdominal aortic aneurysms is closely related to the surgical skill and perioperative management of the surgeons. For patients with lower age and low risk, increasingly more mature surgical techniques and postoperative monitoring and nursing can allow patients to demonstrate better long-term prognoses; therefore, OSR is recommended for young patients (32).

There exists the prospect of treatment in the future. The time of clinical application of the three treatments is different, including 70 years of OSR, 20 years of HSR, and only 25 years of EVAR; thus, the long-term results of EVAR (such as 50 years) are not clear. The management of TAAA via endovascular methods is continually evolving, and reports typically involve cases that happened during the authors’ early learning stages. Hence, there is a possibility that our analysis could be biased owing to the expectation of reduced incidence of adverse events in recent times. Additionally, the open technique, which has been in practice for 70 years, has witnessed significant enhancements such as the introduction of left-sided heart bypass and cold crystalloid renal perfusion (33). Consequently, we can anticipate further advancements and improvements in these approaches in the future (12). However, we believe that EAVR will become the first-line treatment option for TAAAs because, with the continuous progress of endovascular equipment and technology, EVAR not only has the characteristics of low perioperative mortality and low complications but also has low long-term mortality and complications.

There were several limitations to this study. It is worth noting that this meta-analysis has some limitations regarding sample size, comprising only 11 articles and 4,333 patients, which may affect the findings’ reliability. Furthermore, all of the articles reviewed were retrospective studies, which could have reduced their quality. Our systematic review also discovered a crucial concern regarding the reporting of postoperative complications. Specifically, there is a lack of consistency and standardization in the manner in which these complications are reported. Of note, none of the studies under analysis reported all the prespecified outcomes of interest, such as stroke, death, cardiac disease, renal complications, spinal cord ischemia, and pulmonary complications. It is important to exercise caution when comparing groups with small sample sizes that have undergone TAAA repair due to the significant variations in morbidity and mortality rates that can occur depending on the extent of aorta treatment (31).

## Conclusion

EVAR presents a minimally invasive alternative to open TAAA repair. Though there are still many challenges to be addressed when using EVAR to treat all TAAA, it is expected that the continued advancements in patient selection, device design, and perioperative care will drive EVAR's mortality and morbidity rates even lower. According to the results of this study, compared to HSR and OSR, EVAR appears to be a superior approach for treating TAAAs. However, as the included literature is a retrospective study, there is currently no relevant RCT study, and the conclusions in this article are for clinical reference only.

## Data Availability

The original contributions presented in the study are included in the article/Supplementary Materials, further inquiries can be directed to the corresponding author.
