# Supplementary material for: Comparison of efficiency and safety of open surgery, hybrid surgery and endovascular repair for the treatment of thoracoabdominal aneurysms: a systemic review and network meta-analysis
Source: Front Cardiovasc Med. 2023 Dec 15;10:1257628. doi: 10.3389/fcvm.2023.1257628 (PMC10757346; doi:10.3389/fcvm.2023.1257628)
Supplement: Supplementary file 1 [file Datasheet1.pdf]

SUPPLEMENTAL MATERIAL for: *Comparison Efficiency and Safety of Open Surgery, Hybrid Surgery and Endovascular Repair for The Treatment of Thoracoabdominal Aneurysms: A Systemic Review and Network Meta-Analysis*

Contents

Section	Subsection	Pages
Table S1 – PRISMA NMA Checklist	n/a	3-6
Table S2 – Search Strategy	n/a	7-8
Table S3 – Quality assessment of all included studies.	n/a	9
Appendix Figures	**Figure S1** Head-to-Head for comparative all-cause mortality network meta-analysis at 1-month mortality	10
	**Figure S2** Literature summary network plots for comparative survival rate network meta-analysis at 6-month, 1-year, 3-year and 5-year rate	10
	**Figure S3** Head-to-Head for comparative survival rate network meta-analysis at 6-month, 1-year, 3-year and 5-year rate	11
	**Figure S4** Head-to-Head for comparative complication rate network meta-analysis at 1 month rate	12
	**Figure S5** Forest plot comparing 1-month mortality during OSR, HSR, EVAR.	13
	**Figure S6** Global inconsistency plot comparing 1-month mortality during OSR, HSR, EVAR	14
	**Figure S7** Funnel plot comparing 1-month mortality during OSR, HSR, EVAR.	15
	**Figure S8** Trace plot evaluate the MCMC convergence of 1-month mortality during OSR, HSR, EVAR.	16
	**Figure S9** Density plot showing the posterior value of 1-month mortality during OSR, HSR, EVAR	17
	**Figure S10** Contribution plot for all-cause 1-month mortality	18
	**Figure S11** Contribution plot for comparative survival rate network meta-analysis at 6-month, 1-year, 3-year and 5-year rate	19
	**Figure S12** Forest plot comparing 6-month, 1-year, 3-year and 5-year rate during OSR, EVAR.	20

	**Figure S1** Forest plot comparing 6-month, 1-year, 3-year and 5-year rate during HSR, EVAR.	21
	**Figure S14** Forest plot comparing 6-month, 1-year, 3-year and 5-year rate during OSR, HSR.	22
	**Figure S15** Forest plot comparing comparative complication rate during OSR, HSR.	23
	**Figure S16** Forest plot comparing comparative complication rate during OSR, EVAR.	24
	**Figure S17** Forest plot comparing comparative complication rate during HSR, EVAR.	25
Table S4 – Final GRADE Assessment for perioperative mortality comparison between the 3 treatment options	n/a	26-28

Table S1 – PRISMA NMA Checklist

PRISMA NMA Checklist of Items to Include When Reporting A Systematic Review Involving a Network Meta-analysis

Section/Topic	Item #	Checklist Item	Reported on Page #
**TITLE**			
Title	1	Identify the report as a systematic review *incorporating a network meta-analysis (or related form of meta-analysis).*	1
**ABSTRACT**			
Structured summary	2	Provide a structured summary including, as applicable:<br>**Background:** main objectives<br>**Methods:** data sources; study eligibility criteria, participants, and interventions; study appraisal; and *synthesis methods, such as network meta-analysis.*<br>**Results:** number of studies and participants identified; summary estimates with corresponding confidence/credible intervals; *treatment rankings may also be discussed. Authors may choose to summarize pairwise comparisons against a chosen treatment included in their analyses for brevity.*<br>**Discussion/Conclusions:** limitations; conclusions and implications of findings.<br>**Other:** primary source of funding; systematic review registration number with registry name.	2
**INTRODUCTION**			
Rationale	3	Describe the rationale for the review in the context of what is already known, *including mention of why a network meta-analysis has been conducted.*	3-5
Objectives	4	Provide an explicit statement of questions being addressed, with reference to participants, interventions, comparisons, outcomes, and study design (PICOS).	6
**METHODS**			
Protocol and registration	5	Indicate whether a review protocol exists and if and where it can be accessed (e.g., Web address); and, if available, provide registration information, including registration number.	6
Eligibility criteria	6	Specify study characteristics (e.g., PICOS, length of follow-up) and report characteristics (e.g., years considered, language, publication status) used as criteria for eligibility, giving rationale. *Clearly describe eligible treatments included in the treatment network, and note whether any have been clustered or merged into the same node (with justification).*	**7**
Information sources	7	Describe all information sources (e.g., databases with dates of coverage, contact with study authors to identify additional studies) in the search and date last searched.	6
Search	8	Present full electronic search strategy for at least one database, including any limits used, such that it could be repeated.	6
Study selection	9	State the process for selecting studies (i.e., screening, eligibility, included in systematic review, and, if applicable, included in the meta-analysis).	6

Data collection process	10	Describe method of data extraction from reports (e.g., piloted forms, independently, in duplicate) and any processes for obtaining and confirming data from investigators.	7
Data items	11	List and define all variables for which data were sought (e.g., PICOS, funding sources) and any assumptions and simplifications made.	6-7
**Geometry of the network**	**S1**	Describe methods used to explore the geometry of the treatment network under study and potential biases related to it. This should include how the evidence base has been graphically summarized for presentation, and what characteristics were compiled and used to describe the evidence base to readers.	
Risk of bias within individual studies	12	Describe methods used for assessing risk of bias of individual studies (including specification of whether this was done at the study or outcome level), and how this information is to be used in any data synthesis.	7-8
Summary measures	13	State the principal summary measures (e.g., risk ratio, difference in means). *Also describe the use of additional summary measures assessed, such as treatment rankings and surface under the cumulative ranking curve (SUCRA) values, as well as modified approaches used to present summary findings from meta-analyses.*	8
Planned methods of analysis	14	Describe the methods of handling data and combining results of studies for each network meta-analysis. This should include, but not be limited to:<br>• *Handling of multi-arm trials;*<br>• *Selection of variance structure;*<br>• *Selection of prior distributions in Bayesian analyses; and*<br>• *Assessment of model fit.*	8
**Assessment of Inconsistency**	**S2**	Describe the statistical methods used to evaluate the agreement of direct and indirect evidence in the treatment network(s) studied. Describe efforts taken to address its presence when found.	
Risk of bias across studies	15	Specify any assessment of risk of bias that may affect the cumulative evidence (e.g., publication bias, selective reporting within studies).	7-8
Additional analyses	16	Describe methods of additional analyses if done, indicating which were pre-specified. This may include, but not be limited to, the following:<br>• Sensitivity or subgroup analyses;<br>• Meta-regression analyses;<br>• *Alternative formulations of the treatment network; and*<br>• *Use of alternative prior distributions for Bayesian analyses (if applicable).*	/
**RESULTS†**			
Study selection	17	Give numbers of studies screened, assessed for eligibility, and included in the review, with reasons for exclusions at each stage, ideally with a flow diagram.	9
**Presentation of network structure**	**S3**	Provide a network graph of the included studies to enable visualization of the geometry of the treatment network.	

**Summary of network geometry**	**S4**	Provide a brief overview of characteristics of the treatment network. This may include commentary on the abundance of trials and randomized patients for the different interventions and pairwise comparisons in the network, gaps of evidence in the treatment network, and potential biases reflected by the network structure.	
Study characteristics	18	For each study, present characteristics for which data were extracted (e.g., study size, PICOS, follow-up period) and provide the citations.	9
Risk of bias within studies	19	Present data on risk of bias of each study and, if available, any outcome level assessment.	9
Results of individual studies	20	For all outcomes considered (benefits or harms), present, for each study: 1) simple summary data for each intervention group, and 2) effect estimates and confidence intervals. *Modified approaches may be needed to deal with information from larger networks.*	9-11
Synthesis of results	21	Present results of each meta-analysis done, including confidence/credible intervals. *In larger networks, authors may focus on comparisons versus a particular comparator (e.g. placebo or standard care), with full findings presented in an appendix. League tables and forest plots may be considered to summarize pairwise comparisons.* If additional summary measures were explored (such as treatment rankings), these should also be presented.	9-11
**Exploration for inconsistency**	**S5**	Describe results from investigations of inconsistency. This may include such information as measures of model fit to compare consistency and inconsistency models, *P* values from statistical tests, or summary of inconsistency estimates from different parts of the treatment network.	
Risk of bias across studies	22	Present results of any assessment of risk of bias across studies for the evidence base being studied.	9-11
Results of additional analyses	23	Give results of additional analyses, if done (e.g., sensitivity or subgroup analyses, meta-regression analyses, *alternative network geometries studied, alternative choice of prior distributions for Bayesian analyses,* and so forth).	/
**DISCUSSION**			
Summary of evidence	24	Summarize the main findings, including the strength of evidence for each main outcome; consider their relevance to key groups (e.g., healthcare providers, users, and policy-makers).	12-15
Limitations	25	Discuss limitations at study and outcome level (e.g., risk of bias), and at review level (e.g., incomplete retrieval of identified research, reporting bias). *Comment on the validity of the assumptions, such as transitivity and consistency. Comment on any concerns regarding network geometry (e.g., avoidance of certain comparisons).*	15
Conclusions	26	Provide a general interpretation of the results in the context of other evidence, and implications for future research.	15-16
**FUNDING**			
Funding	27	Describe sources of funding for the systematic review and other support (e.g., supply of data); role of funders for the systematic review. This should also include information regarding whether funding has been received from manufacturers of treatments in	1

	the network and/or whether some of the authors are content experts with professional conflicts of interest that could affect use of treatments in the network.	

PICOS = population, intervention, comparators, outcomes, study design.
* Text in italics indicateS wording specific to reporting of network meta-analyses that has been added to guidance from the PRISMA statement.
† Authors may wish to plan for use of appendices to present all relevant information in full detail for items in this section.

Table S2 – Search Strategy

**PubMed**<br>The database was searched on December 24th, 2022, n=853.<br><br>Search Strategy:<br><br>1.(endovascular repair [Title/Abstract]) AND (thoracoabdominal aortic aneurysms [Title/Abstract])<br><br>2.(hybrid surgery repair [Title/Abstract]) AND (thoracoabdominal aortic aneurysms [Title/Abstract])<br><br>3.(open surgical repair [Title/Abstract]) AND (thoracoabdominal aortic aneurysms [Title/Abstract])
**EMBASE**<br>The database was searched on December 24th, 2022, n=1319.<br><br>Search Strategy:<br><br>1.('thoracoabdominal aortic aneurysm':ti,ab,kw) AND ('endovascular repair':ti,ab,kw)<br><br>2.('thoracoabdominal aortic aneurysm':ti,ab,kw) AND ('hybrid surgery repair':ti,ab,kw)<br><br>3.('thoracoabdominal aortic aneurysm':ti,ab,kw) AND ('open surgical repair':ti,ab,kw)
**Web of Science**<br><br>The database was searched on December 24th, 2022, n=884.<br><br>Search Strategy:<br><br>#1: TOPIC: (“thoracoabdominal aortic aneurysm”)<br><br>#2: TOPIC: (“endovascular repair”)<br><br>#3: TOPIC: (“hybrid surgery repair”)<br><br>#4: TOPIC: (“open surgical repair”)<br><br>1.#1 AND #2<br><br>2.#1AND #3<br><br>3.#1AND #4
**Scopus**<br>The database was searched on December 24th, 2022, n=167<br><br>Search Strategy:<br><br>1.TITLE-ABS-KEY ((“thoracoabdominal aortic aneurysm”) and (“endovascular repair”))<br>2.TITLE-ABS-KEY ((“thoracoabdominal aortic aneurysm”)and (“hybrid surgery repair”))<br>3.TITLE-ABS-KEY ((“thoracoabdominal aortic aneurysm”)and (“open surgical repair”))

ScienceDirect
The database was searched on December 24th, 2022, n=1583

Search Strategy:

1.Title, abstract, keywords: ((“thoracoabdominal aortic aneurysm”) and (“endovascular repair”))
2.Title, abstract, keywords: ((“thoracoabdominal aortic aneurysm”)and (“hybrid surgery repair”))
3.Title, abstract, keywords: ((“thoracoabdominal aortic aneurysm”)and (“open surgical repair”))

Cochrane Library
The database was searched on December 24th, 2022, n=99.

Search Strategy:

1.(“thoracoabdominal aortic aneurysm”): ti,ab,kw AND (“endovascular repair”): ti,ab,kw

2.(“thoracoabdominal aortic aneurysm”): ti,ab,kw AND (“hybrid surgery repair”): ti,ab,kw

3.(“thoracoabdominal aortic aneurysm”): ti,ab,kw AND (“open surgical repair”): ti,ab,kw

Clinical Trail
The database was searched on December 24th, 2022, n=47.

Search Strategy:

Condition or disease: Thoracoabdominal Aortic Aneurysm: Other terms : repair

China National Knowledge Infrastructure (CNKI)
The database was searched on December 24th, 2022, n=239.

Search Strategy:

1. 胸腹主动脉瘤 (13563)
2. 治疗 (10549898)
3. 1 and 2 (239)

Table S3 – Quality assessment of all included studies.

	Study	Selection					Outcome			Total score
		Exposed cohort[a]	Nonexposed cohort[b]	Ascertainment of exposure	Outcome of interest[c]	Comparability[d]	Assessment of outcome	Length of follow-up[e]	Adequacy of follow-up	
2007	Chiesa	*	*	*	*		*			5
2010	Patel	*	*	*	*		*	*	*	7
2015	Ci	*	*	*	*		*		*	6
2016	Benrashid	*	*	*	*		*	*		6
2016	Ferrer	*	*	*	*	**	*			7
2016	Feng	*	*	*	*		*	*	*	7
2018	Locham	*	*	*	*		*			5
2018	Geisbüsch	*	*	*	*		*			5
2018	Bertoglio	*	*	*	*	*	*			6
2019	Kang	*	*	*	*		*	*	*	7
2019	Arnaoutakis	*	*	*	*		*	*	*	7

Note: [a] Representativeness of the exposed cohort; [b] Selection of the non-exposed cohort; [c] Demonstration that outcome of interest was not present at start of study; [d] Comparability of cohorts on the basis of the design or analysis; [e] Was follow-up long enough for outcomes to occur.

1-month mortality

EVAR	3.27 (1.42, 5.73)	2.68 (1.42, 4.46)
0.31 (0.17, 0.70)	HSR	0.82 (0.50, 1.51)
0.37 (0.22, 0.71)	1.22 (0.66, 1.98)	OSR

Figure S1 Head-to-Head for comparative all-cause mortality network meta-analysis at 1-month mortality (4222 patients across 11 studies; open surgery 2222 patients, HSR 537 patients, and EVAR 1574 patients). 1-month mortality result are presented as risk ratio (95% confidence interval [CI]). OSR = Open Surgical Repair; HSR = Hybrid Surgery Repair; EVAR = Endovascular Repair.

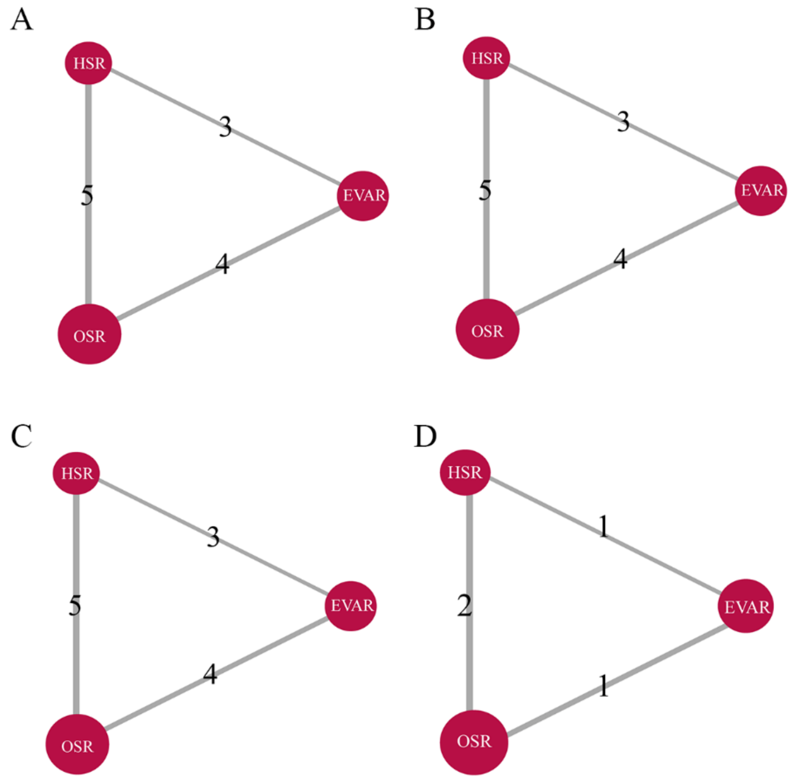

Figure S2 Literature summary network plots for comparative survival rate network meta-analysis at 6-month, 1-year, 3-year and 5-year rate (4222 patients across 11 studies) in studies providing comparative outcomes between methods of Thoracoabdominal aortic aneurysms (TAAA) repair. The size of each red node corresponds to the number of study arms included for a treatment across all comparisons. The width of each grey line corresponds to the number of studies comparing the two interventions directly. OSR = Open Surgical Repair; HSR = Hybrid Surgery Repair; EVAR = Endovascular Repair.

6-month survival rate

EVAR	0.28 (0.06, 1.26)	0.36 (0.08, 1.52)
3.58 (0.79, 16.39)	HSR	1.28 (0.41, 3.84)
2.76 (0.66, 12.07)	0.78 (0.26, 2.42)	OSR

1-year survival rate

EVAR	0.41 (0.11, 1.55)	0.57 (0.16, 2.14)
2.43 (0.64, 8.91)	HSR	1.40 (0.51, 3.80)
1.75 (0.47, 6.45)	0.72 (0.26, 1.97)	OSR

3-year survival rate

EVAR	0.57 (0.07, 5.41)	1.01 (0.12, 8.87)
1.74 (0.18, 14.25)	HSR	1.73 (0.32, 8.85)
0.99 (0.11, 8.08)	0.58 (0.11, 3.11)	OSR

5-year survival rate

EVAR	0.48 (0.00, 45.48)	2.62 (0.03, 239.89)
2.07 (0.02, 204.17)	HSR	5.40 (0.18, 173.55)
0.38 (0.00, 32.18)	0.19 (0.01, 5.57)	OSR

Figure S3 Head-to-Head for comparative survival rate network meta-analysis at 6-month, 1-year, 3-year and 5-year rate (4222 patients across 11 studies; open surgery 2222 patients, HSR 537 patients, and EVAR 1574 patients). The results are presented as risk ratio (95% confidence interval [CI]). OSR = Open Surgical Repair; HSR = Hybrid Surgery Repair; EVAR = Endovascular Repair.

Cardiac Complications

EVAR	1.34 (0.20, 10.17)	2.47 (0.49, 14.15)
0.75 (0.10, 5.09)	HSR	1.88 (0.32, 12.22)
0.40 (0.07, 2.05)	0.53 (0.08, 3.15)	OSR

Pulmonary Complications

EVAR	3.36 (0.43, 29.86)	7.48 (2.12, 64.02)
0.30 (0.03, 2.32)	HSR	2.19 (0.58, 18.22)
0.13 (0.02, 0.47)	0.46 (0.05, 1.72)	OSR

Renal Complications

EVAR	4.98 (2.32, 12.75)	2.91 (1.53, 6.45)
0.20 (0.08, 0.43)	HSR	0.58 (0.29, 1.18)
0.34 (0.16, 0.65)	1.71 (0.85, 3.42)	OSR

Spinal Cord Ischemia

EVAR	0.49 (0.14, 1.33)	1.09 (0.44, 2.57)
2.06 (0.75, 7.21)	HSR	2.25 (0.90, 7.16)
0.92 (0.39, 2.27)	0.45 (0.14, 1.11)	OSR

Stroke

EVAR	1.56 (0.38, 5.11)	2.32 (0.88, 5.69)
0.64 (0.20, 2.60)	HSR	1.52 (0.55, 4.74)
0.43 (0.18, 1.14)	0.66 (0.21, 1.80)	OSR

Figure S4 Head-to-Head for comparative complication rate network meta-analysis at 1 month rate (4222 patients across 11 studies; open surgery 2222 patients, HSR 537 patients, and EVAR 1574 patients). The results are presented as risk ratio (95% confidence interval [CI]). OSR = Open Surgical Repair; HSR = Hybrid Surgery Repair; EVAR = Endovascular Repair.

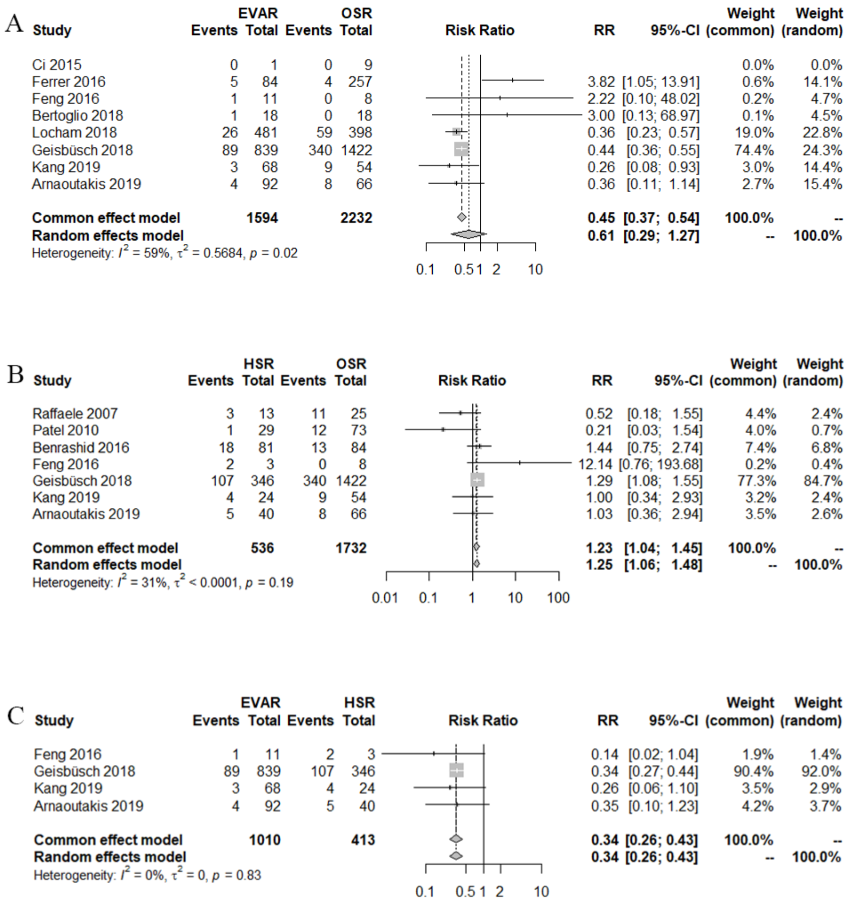

Figure S5 Forest plot comparing 1-month mortality during OSR, HSR, EVAR. The pooled risk ratios with 95% confidence intervals (CIs) were calculated using fixed-effects models. (OSR = Open Surgical Repair; HSR = Hybrid Surgery Repair; EVAR = Endovascular Repair.)

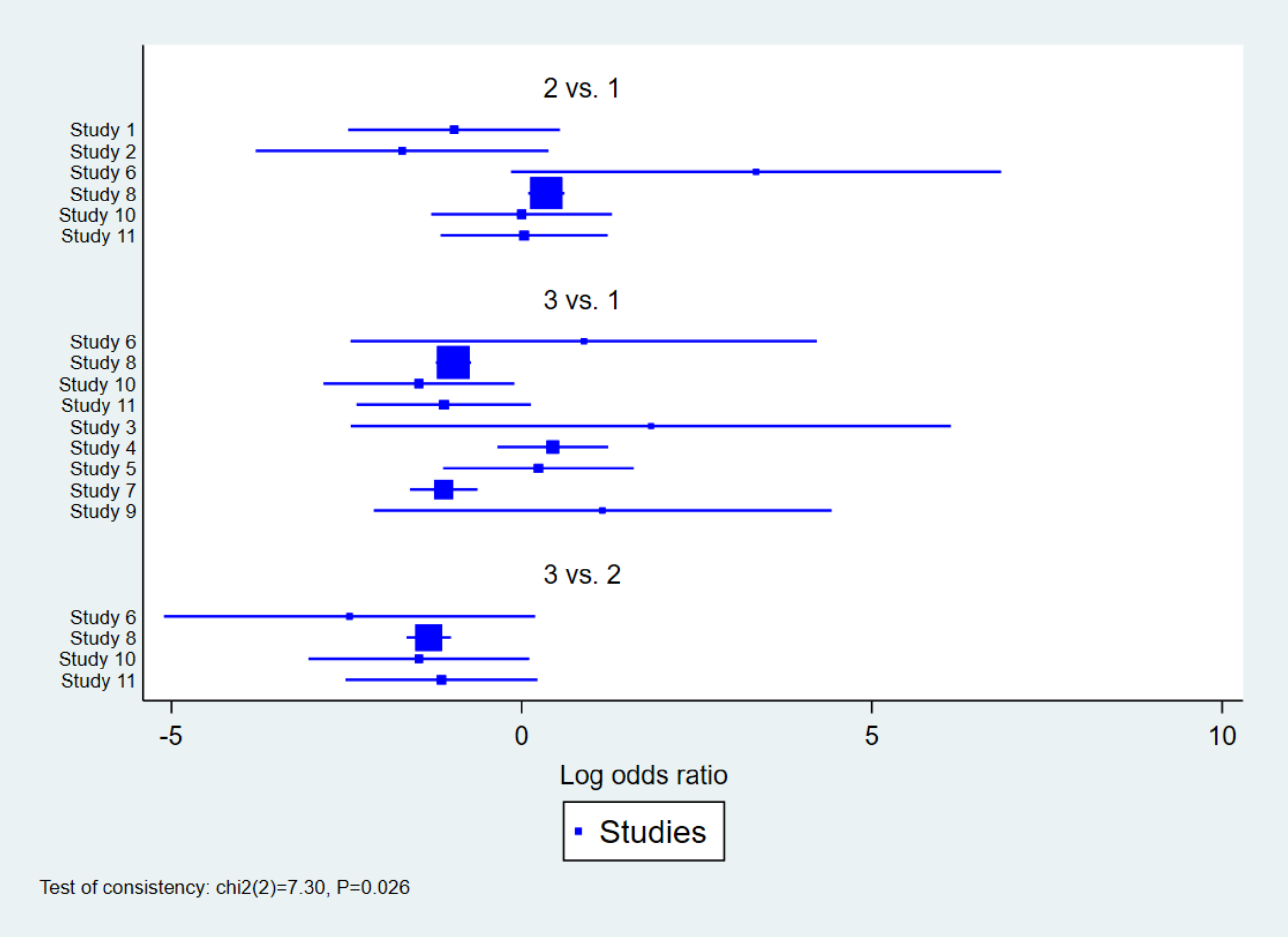

Figure S6 Global inconsistency plot comparing 1-month mortality during OSR, HSR, EVAR. P > 0.05 shows the inconsistency test is not significant, indicating that the consistency model can be used for analysis. (1: OSR = Open Surgical Repair; 2: HSR = Hybrid Surgery Repair; 3: EVAR = Endovascular Repair.)

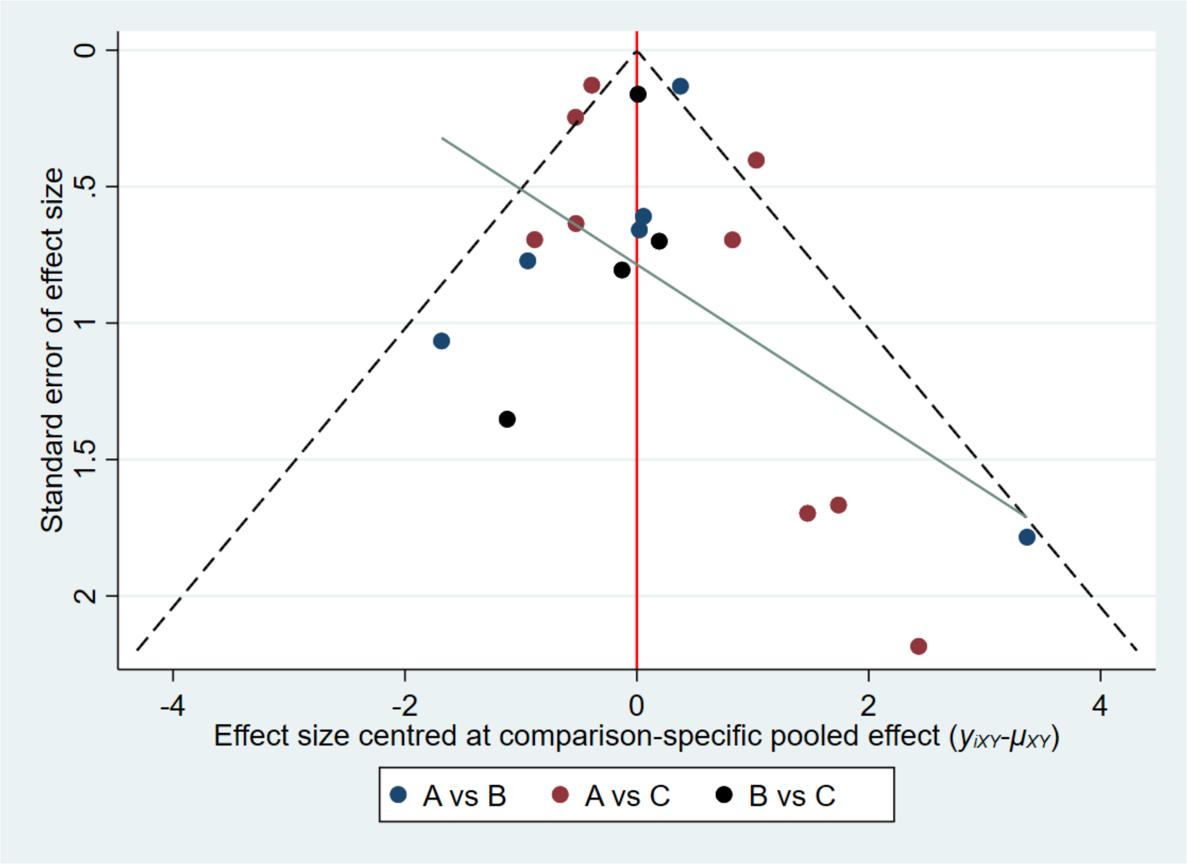

Figure S7 Funnel plot comparing 1-month mortality during OSR, HSR, EVAR. The study with small sample size has a large number and low precision, and the distribution is symmetrically arranged at the bottom of the funnel diagram; the study with large sample size has high precision and is distributed at the top of the funnel diagram and concentrated in the middle. The funnel diagram shows asymmetry and biased distribution means the studies have publication bias. (A:OSR = Open Surgical Repair; B:HSR = Hybrid Surgery Repair; C:EVAR = Endovascular Repair.)

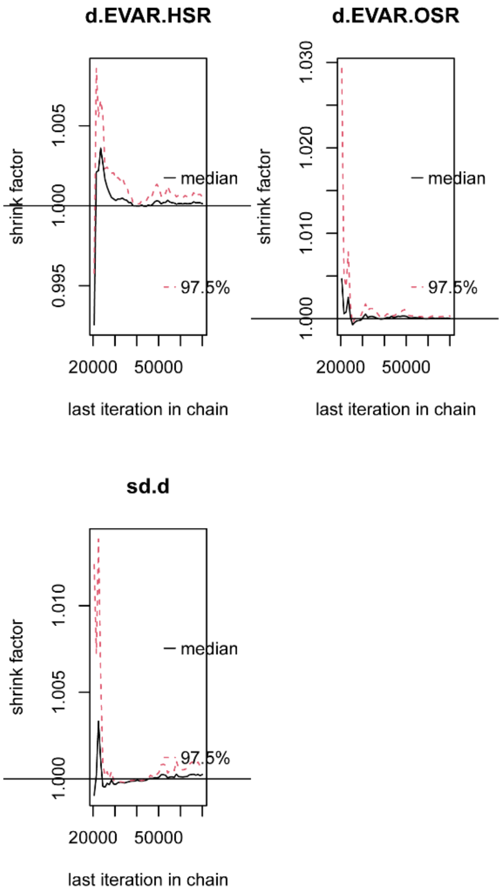

Figure S8 Trace plot evaluate the MCMC convergence of 1-month mortality during OSR, HSR, EVAR. The horizontal axis shows the number of iterations and the vertical axis shows the number of iterations after the parameter. Check the distribution value, when the MCMC reaches a steady state, the simulated value of the parameter, will fluctuate up and down by the same magnitude near the mean. (MCMC=Markov Chain Monte Carlo, OSR = Open Surgical Repair; HSR = Hybrid Surgery Repair; EVAR = Endovascular Repair).

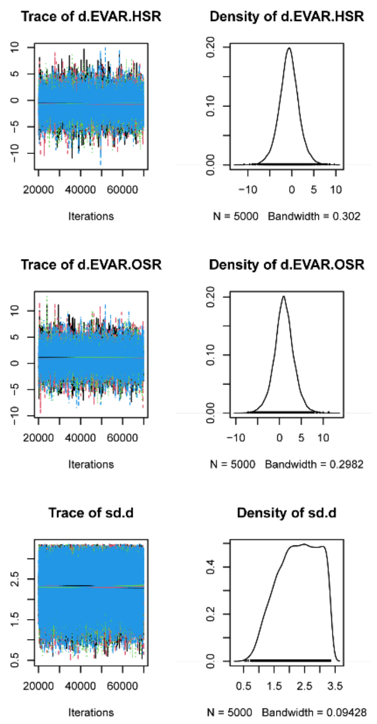

Figure S9 Density plot showing the posterior value of 1-month mortality during OSR, HSR, EVAR, which is used to diagnose the convergence range of the model. (OSR = Open Surgical Repair; HSR = Hybrid Surgery Repair; EVAR = Endovascular Repair).

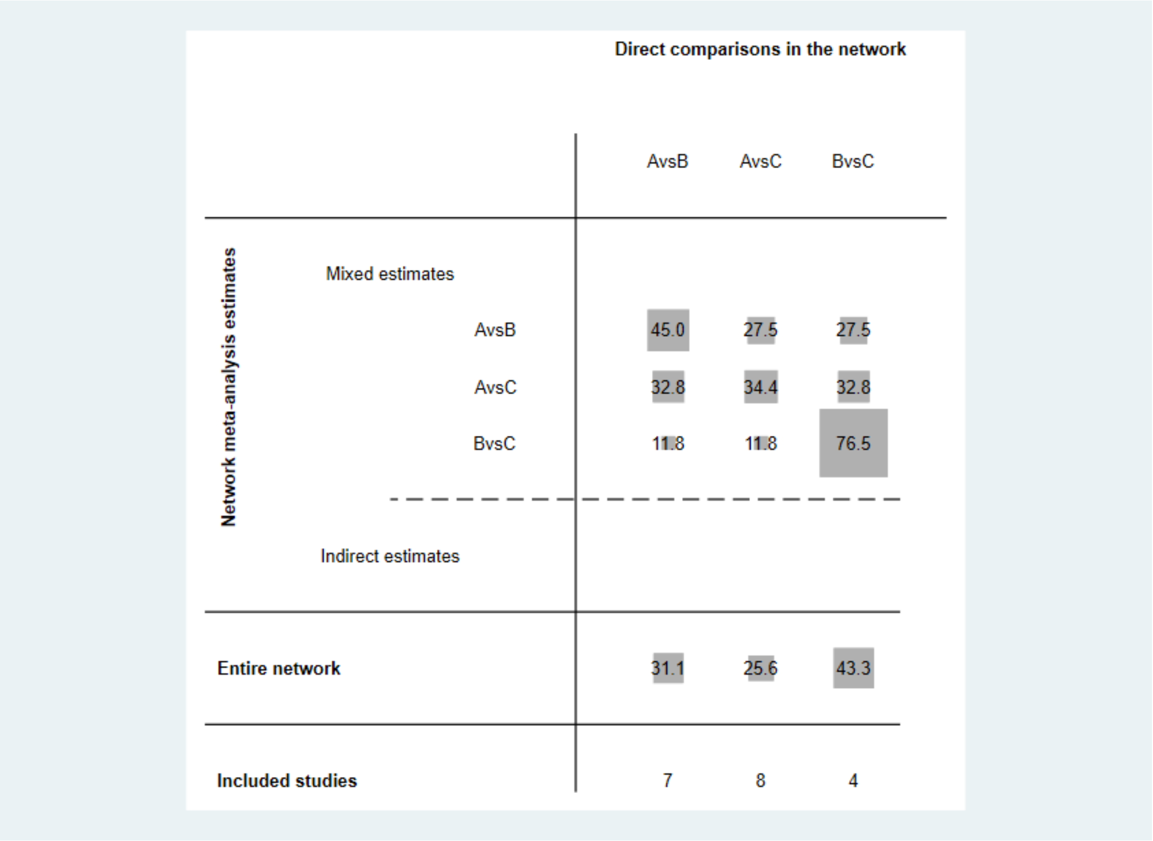

Figure S10 Contribution plot for all-cause 1-month mortality (4222 patients across 11 studies) in studies providing comparative outcomes between methods of Thoracoabdominal aortic aneurysms (TAAA) repair. The size of each square is proportional to the weight attached to each direct summary effect (horizontal axis) for the estimation of each network summary effects (vertical axis). The numbers re-express the weights as percentages. (A:OSR = Open Surgical Repair; B:HSR = Hybrid Surgery Repair; C:EVAR = Endovascular Repair).

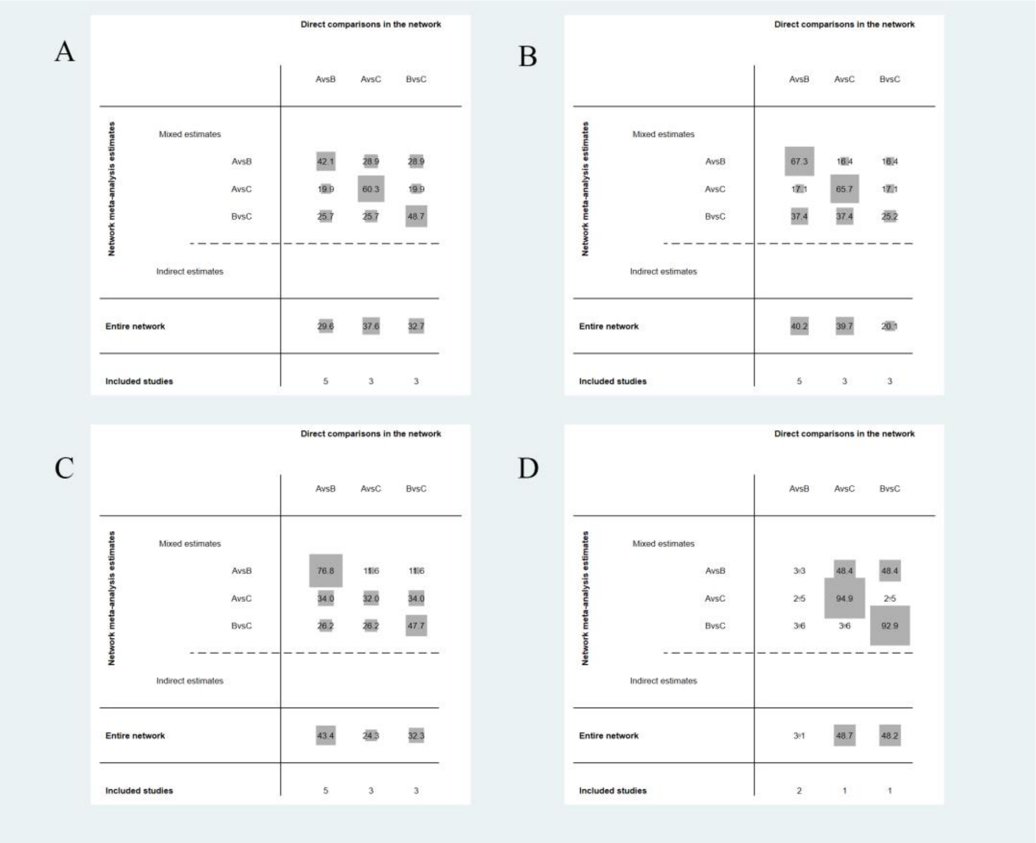

Figure S11 Contribution plot for comparative survival rate network meta-analysis at 6-month, 1-year, 3-year and 5-year rate (4222 patients across 11 studies) in studies providing comparative outcomes between methods of Thoracoabdominal aortic aneurysms (TAAA) repair. The size of each square is proportional to the weight attached to each direct summary effect (horizontal axis) for the estimation of each network summary effects (vertical axis). The numbers re-express the weights as percentages. (A:OSR = Open Surgical Repair; B:HSR = Hybrid Surgery Repair; C:EVAR = Endovascular Repair).

Study or Subgroup	EVAR Events	EVAR Total	HSR Events	HSR Total	Weight	Risk Ratio M-H, Fixed, 95% CI	Year
**2.12.2 6 months**							
feng 2016	11	11	3	3	2.0%	1.00 [0.68, 1.48]	2016
Arnaoutakis 2019	85	92	35	40	18.8%	1.06 [0.93, 1.20]	2019
Kang 2019	62	68	18	24	10.3%	1.22 [0.95, 1.55]	2019
**Subtotal (95% CI)**		**171**		**67**	**31.1%**	**1.10 [0.98, 1.24]**	
Total events	158		56				
Heterogeneity: Chi² = 1.31, df = 2 (P = 0.52); I² = 0%							
Test for overall effect: Z = 1.68 (P = 0.09)							
**2.12.3 1 year**							
feng 2016	11	11	3	3	2.0%	1.00 [0.68, 1.48]	2016
Kang 2019	60	68	16	24	9.1%	1.32 [0.98, 1.78]	2019
Arnaoutakis 2019	78	92	34	40	18.3%	1.00 [0.85, 1.17]	2019
**Subtotal (95% CI)**		**171**		**67**	**29.4%**	**1.10 [0.96, 1.26]**	
Total events	149		53				
Heterogeneity: Chi² = 3.22, df = 2 (P = 0.20); I² = 38%							
Test for overall effect: Z = 1.33 (P = 0.18)							
**2.12.4 3 years**						Modified by random-effect model	
feng 2016	11	11	3	3	4.9%	1.00 [0.68, 1.48]	2016
Arnaoutakis 2019	66	92	33	40	14.7%	0.87 [0.72, 1.05]	2019
Kang 2019	51	68	12	24	4.2%	1.50 [0.98, 2.29]	2019
**Subtotal (95% CI)**		**171**		**67**	**23.9%**	**1.05 [0.75, 1.48]**	
Total events	128		48				
Heterogeneity: Tau² = 0.06; Chi² = 6.30, df = 2 (P = 0.04); I² = 68%							
Test for overall effect: Z = 0.31 (P = 0.76)							
**2.12.5 5 years**							
Arnaoutakis 2019	48	92	24	40	12.9%	0.87 [0.63, 1.20]	2019
**Subtotal (95% CI)**		**92**		**40**	**12.9%**	**0.87 [0.63, 1.20]**	
Total events	48		24				
Heterogeneity: Not applicable							
Test for overall effect: Z = 0.86 (P = 0.39)							
**Total (95% CI)**		**605**		**241**	**100.0%**	**1.06 [0.97, 1.15]**	
Total events	483		181				
Heterogeneity: Chi² = 12.27, df = 9 (P = 0.20); I² = 27%							
Test for overall effect: Z = 1.30 (P = 0.19)							
Test for subgroup differences: Chi² = 2.12, df = 3 (P = 0.55), I² = 0%							

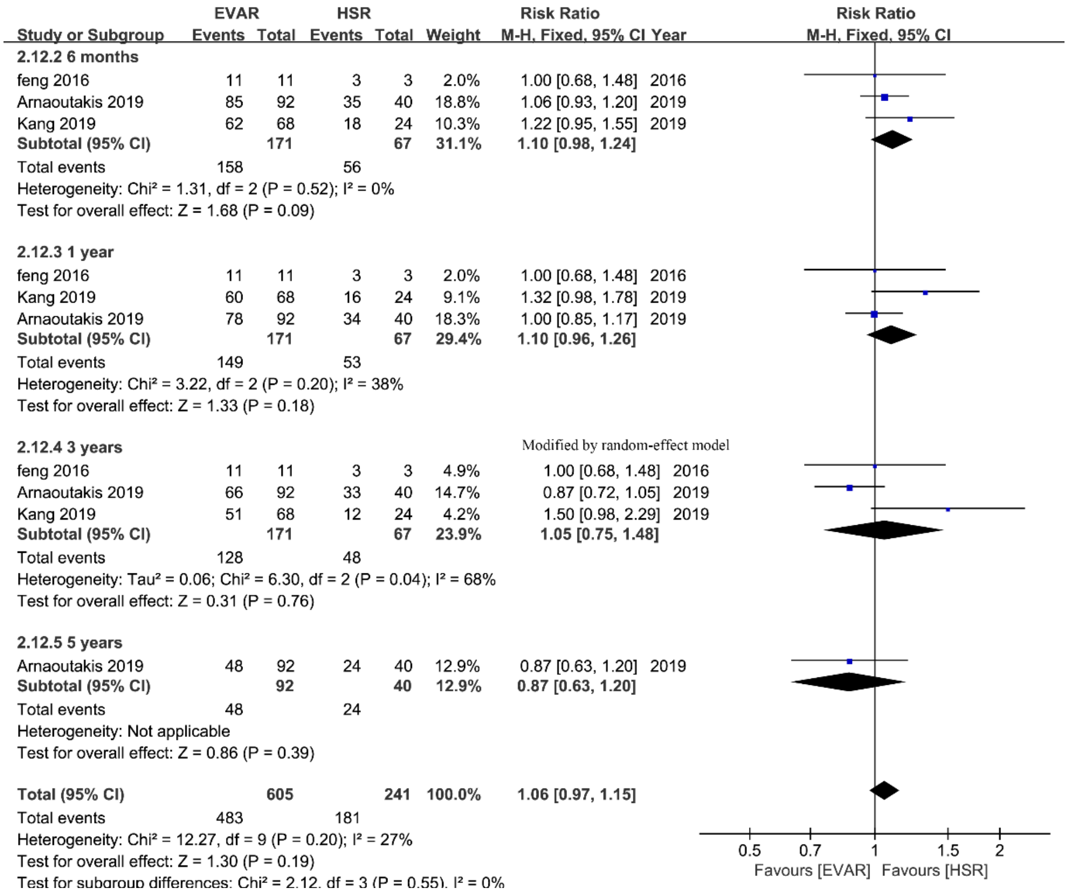

Figure S12 Forest plot comparing 6-month, 1-year, 3-year and 5-year rate during OSR, EVAR. The pooled risk ratios with 95% confidence intervals (CIs) were calculated using fixed-effects models and random-effects models. (OSR = Open Surgical Repair; EVAR = Endovascular Repair.)

Study or Subgroup	EVAR Events	EVAR Total	OSR Events	OSR Total	Weight	Risk Ratio M-H, Fixed, 95% CI	Year
**3.12.2 6 months**						Modified by random-effect model	
feng 2016	11	11	8	8	5.6%	1.00 [0.82, 1.22]	2016
Ferrer 2016	59	65	59	65	14.8%	1.00 [0.90, 1.12]	2016
Arnaoutakis 2019	85	92	48	66	8.2%	1.27 [1.08, 1.49]	2019
Kang 2019	62	68	45	54	10.1%	1.09 [0.95, 1.26]	2019
**Subtotal (95% CI)**		**236**		**193**	**38.7%**	**1.08 [0.96, 1.22]**	
Total events	217		160				
Heterogeneity: Tau² = 0.01; Chi² = 7.53, df = 3 (P = 0.06); I² = 60%							
Test for overall effect: Z = 1.35 (P = 0.18)							
**3.12.3 1 year**							
feng 2016	11	11	8	8	1.8%	1.00 [0.82, 1.22]	2016
Ferrer 2016	59	65	59	65	10.8%	1.00 [0.90, 1.12]	2016
Arnaoutakis 2019	78	92	48	66	10.2%	1.17 [0.98, 1.38]	2019
Kang 2019	60	68	42	54	8.6%	1.13 [0.96, 1.34]	2019
**Subtotal (95% CI)**		**236**		**193**	**31.4%**	**1.09 [1.00, 1.19]**	
Total events	208		157				
Heterogeneity: Chi² = 3.95, df = 3 (P = 0.27); I² = 24%							
Test for overall effect: Z = 2.05 (P = 0.04)							
**3.12.4 3 years**							
feng 2016	54	65	55	65	10.1%	0.98 [0.84, 1.14]	2016
Ferrer 2016	11	11	8	8	1.8%	1.00 [0.82, 1.22]	2016
Arnaoutakis 2019	66	92	41	66	8.7%	1.15 [0.92, 1.45]	2019
Kang 2019	51	68	38	54	7.8%	1.07 [0.85, 1.33]	2019
**Subtotal (95% CI)**		**236**		**193**	**28.3%**	**1.06 [0.95, 1.18]**	
Total events	182		142				
Heterogeneity: Chi² = 1.85, df = 3 (P = 0.60); I² = 0%							
Test for overall effect: Z = 1.04 (P = 0.30)							
**3.12.5 5 years**							
Arnaoutakis 2019	48	92	39	66	8.3%	0.88 [0.67, 1.17]	2019
**Subtotal (95% CI)**		**92**		**66**	**8.3%**	**0.88 [0.67, 1.17]**	
Total events	48		39				
Heterogeneity: Not applicable							
Test for overall effect: Z = 0.87 (P = 0.38)							
**Total (95% CI)**		**800**		**645**	**100.0%**	**1.07 [1.02, 1.13]**	
Total events	655		498				
Heterogeneity: Chi² = 13.89, df = 12 (P = 0.31); I² = 14%							
Test for overall effect: Z = 2.61 (P = 0.009)							
Test for subgroup differences: Chi² = 2.71, df = 3 (P = 0.44), I² = 0%							

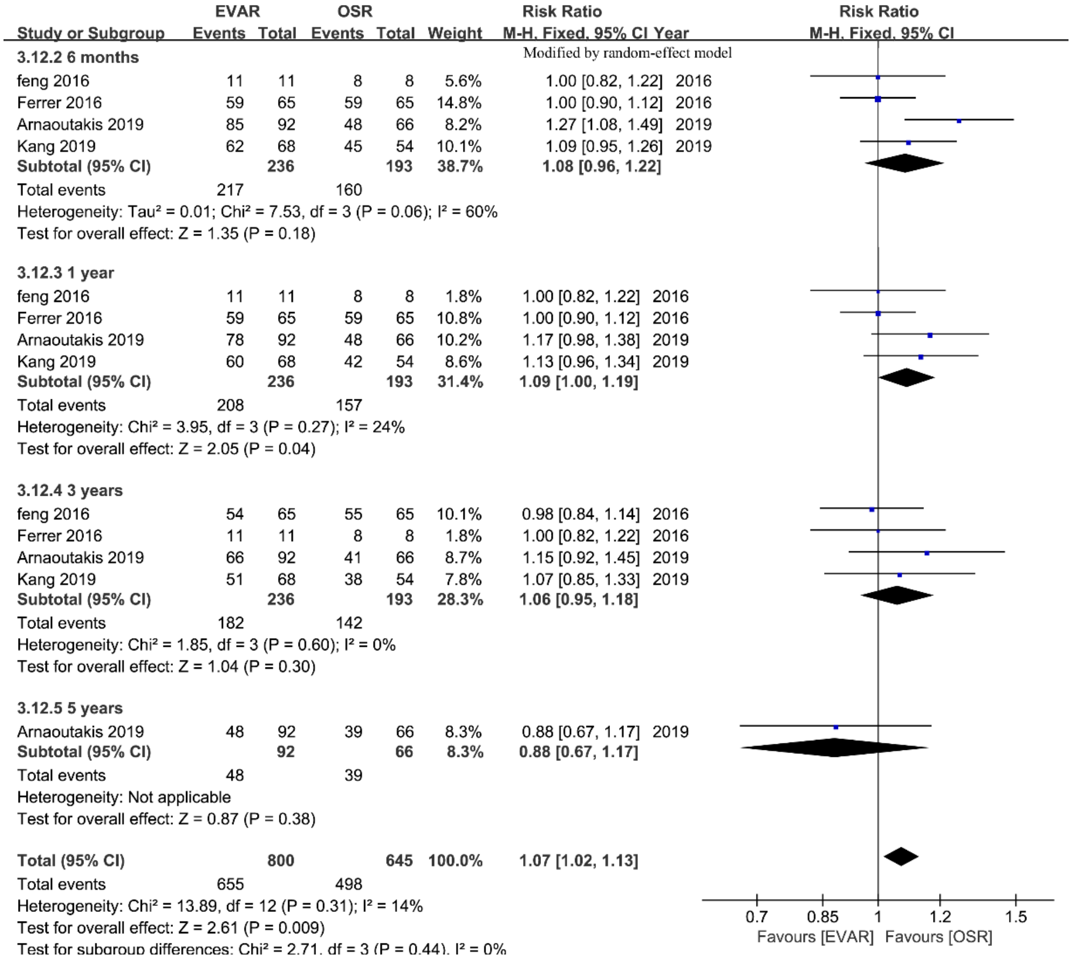

Figure S13 Forest plot comparing 6-month, 1-year, 3-year and 5-year rate during HSR, EVAR. The pooled risk ratios with 95% confidence intervals (CIs) were calculated using fixed-effects models and random-effects models. (HSR = Hybrid Surgery Repair; EVAR = Endovascular Repair.)

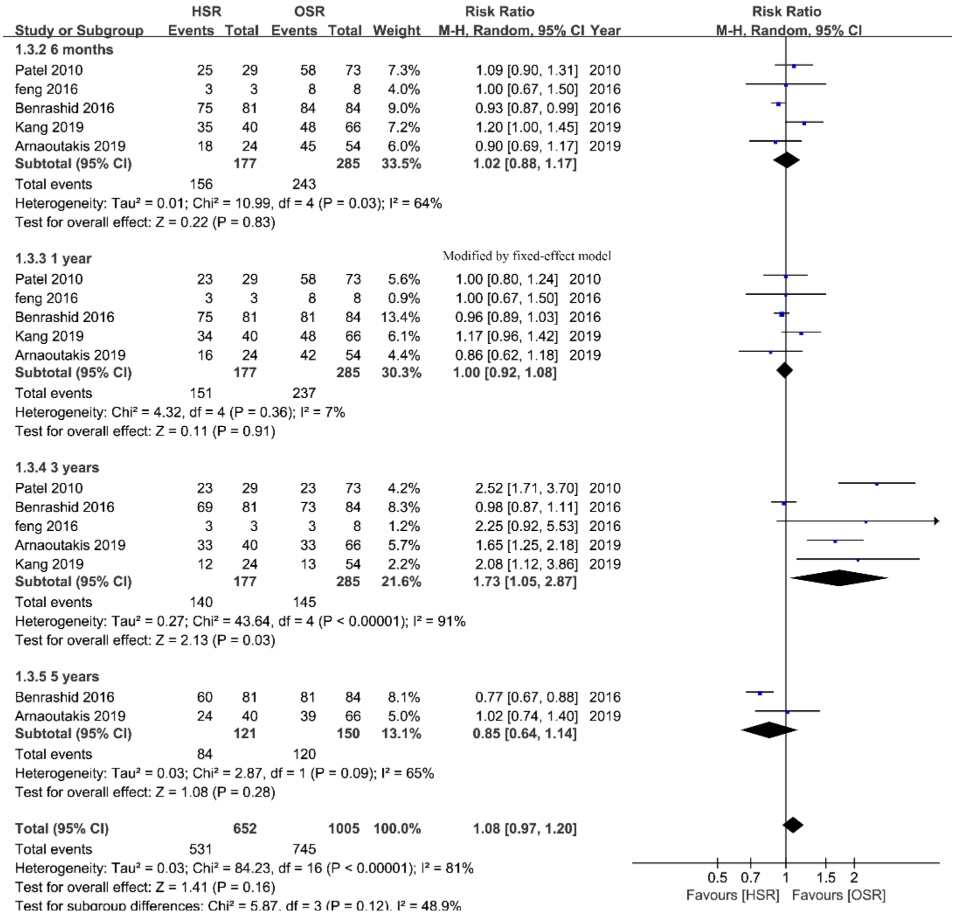

Study or Subgroup	HSR Events	HSR Total	OSR Events	OSR Total	Weight	Risk Ratio M-H, Random, 95% CI	Year
**1.3.2 6 months**							
Patel 2010	25	29	58	73	7.3%	1.09 [0.90, 1.31]	2010
feng 2016	3	3	8	8	4.0%	1.00 [0.67, 1.50]	2016
Benrashid 2016	75	81	84	84	9.0%	0.93 [0.87, 0.99]	2016
Kang 2019	35	40	48	66	7.2%	1.20 [1.00, 1.45]	2019
Arnaoutakis 2019	18	24	45	54	6.0%	0.90 [0.69, 1.17]	2019
**Subtotal (95% CI)**		**177**		**285**	**33.5%**	**1.02 [0.88, 1.17]**	
Total events	156		243				
Heterogeneity: Tau² = 0.01; Chi² = 10.99, df = 4 (P = 0.03); I² = 64%							
Test for overall effect: Z = 0.22 (P = 0.83)							
**1.3.3 1 year**						Modified by fixed-effect model	
Patel 2010	23	29	58	73	5.6%	1.00 [0.80, 1.24]	2010
feng 2016	3	3	8	8	0.9%	1.00 [0.67, 1.50]	2016
Benrashid 2016	75	81	81	84	13.4%	0.96 [0.89, 1.03]	2016
Kang 2019	34	40	48	66	6.1%	1.17 [0.96, 1.42]	2019
Arnaoutakis 2019	16	24	42	54	4.4%	0.86 [0.62, 1.18]	2019
**Subtotal (95% CI)**		**177**		**285**	**30.3%**	**1.00 [0.92, 1.08]**	
Total events	151		237				
Heterogeneity: Chi² = 4.32, df = 4 (P = 0.36); I² = 7%							
Test for overall effect: Z = 0.11 (P = 0.91)							
**1.3.4 3 years**							
Patel 2010	23	29	23	73	4.2%	2.52 [1.71, 3.70]	2010
Benrashid 2016	69	81	73	84	8.3%	0.98 [0.87, 1.11]	2016
feng 2016	3	3	3	8	1.2%	2.25 [0.92, 5.53]	2016
Arnaoutakis 2019	33	40	33	66	5.7%	1.65 [1.25, 2.18]	2019
Kang 2019	12	24	13	54	2.2%	2.08 [1.12, 3.86]	2019
**Subtotal (95% CI)**		**177**		**285**	**21.6%**	**1.73 [1.05, 2.87]**	
Total events	140		145				
Heterogeneity: Tau² = 0.27; Chi² = 43.64, df = 4 (P < 0.00001); I² = 91%							
Test for overall effect: Z = 2.13 (P = 0.03)							
**1.3.5 5 years**							
Benrashid 2016	60	81	81	84	8.1%	0.77 [0.67, 0.88]	2016
Arnaoutakis 2019	24	40	39	66	5.0%	1.02 [0.74, 1.40]	2019
**Subtotal (95% CI)**		**121**		**150**	**13.1%**	**0.85 [0.64, 1.14]**	
Total events	84		120				
Heterogeneity: Tau² = 0.03; Chi² = 2.87, df = 1 (P = 0.09); I² = 65%							
Test for overall effect: Z = 1.08 (P = 0.28)							
**Total (95% CI)**		**652**		**1005**	**100.0%**	**1.08 [0.97, 1.20]**	
Total events	531		745				
Heterogeneity: Tau² = 0.03; Chi² = 84.23, df = 16 (P < 0.00001); I² = 81%							
Test for overall effect: Z = 1.41 (P = 0.16)							
Test for subgroup differences: Chi² = 5.87, df = 3 (P = 0.12), I² = 48.9%							

Figure S14 Forest plot comparing 6-month, 1-year, 3-year and 5-year rate during OSR, HSR. The pooled risk ratios with 95% confidence intervals (CIs) were calculated using fixed-effects models and random-effects models. (OSR = Open Surgical Repair; HSR = Hybrid Surgery Repair.)

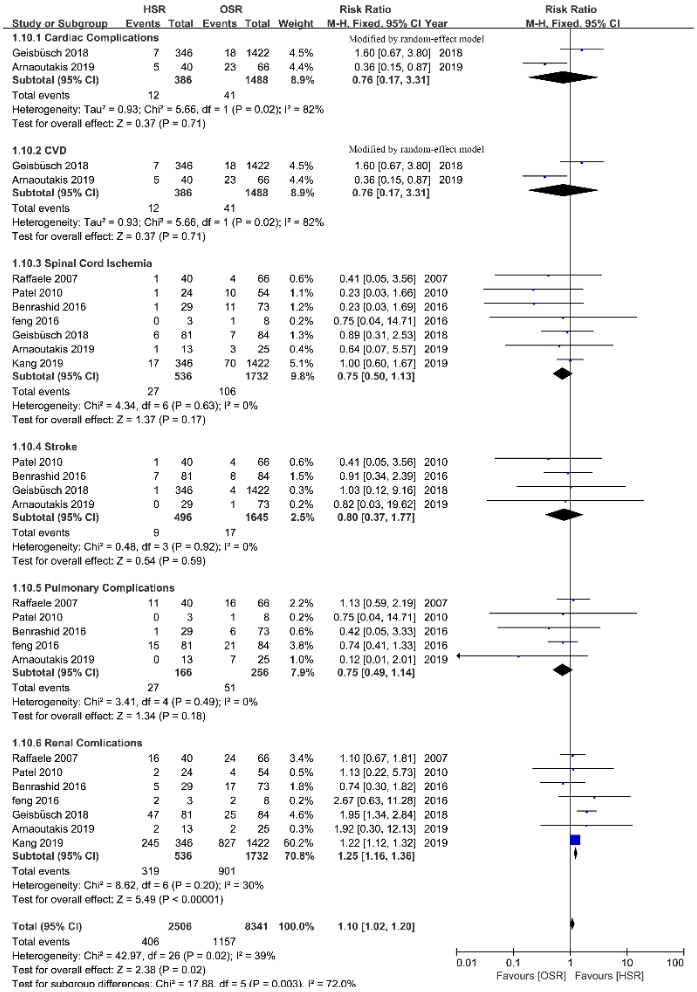

Figure S15 Forest plot comparing comparative complication rate during OSR, HSR. The pooled risk ratios with 95% confidence intervals (CIs) were calculated using fixed-effects models and random-effects models. (OSR = Open Surgical Repair; HSR = Hybrid Surgery Repair.)

	EVAR		OSR			Risk Ratio	
Study or Subgroup	Events	Total	Events	Total	Weight	M-H, Fixed, 95% CI	Year
**3.13.1 Cardiac Complications**							
Geisbüsch 2018	1	839	4	1422	0.3%	0.42 [0.05, 3.78]	2018
Locham 2018	11	481	26	398	2.5%	0.35 [0.18, 0.70]	2018
Arnaoutakis 2019	5	92	23	66	2.3%	0.16 [0.06, 0.39]	2019
**Subtotal (95% CI)**		**1412**		**1886**	**5.1%**	**0.26 [0.16, 0.45]**	
Total events	17		53				
Heterogeneity: Chi² = 2.09, df = 2 (P = 0.35); I² = 4%							
Test for overall effect: Z = 4.96 (P < 0.00001)							
**3.13.2 Spinal Cord Ischemia**						Modified by random-effect model	
Ci 2015	0	1	0	9		Not estimable	2015
Ferrer 2016	0	11	1	8	0.8%	0.25 [0.01, 5.45]	2016
feng 2016	6	65	7	65	4.2%	0.86 [0.30, 2.41]	2016
Geisbüsch 2018	1	18	0	18	0.8%	3.00 [0.13, 69.09]	2018
Bertoglio 2018	48	839	70	1422	7.8%	1.16 [0.81, 1.66]	2018
Locham 2018	14	481	31	398	6.4%	0.37 [0.20, 0.69]	2018
Kang 2019	8	68	10	54	5.1%	0.64 [0.27, 1.50]	2019
Arnaoutakis 2019	17	92	10	66	5.8%	1.22 [0.60, 2.49]	2019
**Subtotal (95% CI)**		**1575**		**2040**	**30.9%**	**0.80 [0.50, 1.27]**	
Total events	94		129				
Heterogeneity: Tau² = 0.17; Chi² = 12.34, df = 6 (P = 0.05); I² = 51%							
Test for overall effect: Z = 0.95 (P = 0.34)							
**3.13.3 Stroke**							
Geisbüsch 2018	1	839	4	1422	0.3%	0.42 [0.05, 3.78]	2018
Locham 2018	11	481	26	398	2.5%	0.35 [0.18, 0.70]	2018
Arnaoutakis 2019	4	92	4	66	0.4%	0.72 [0.19, 2.77]	2019
**Subtotal (95% CI)**		**1412**		**1886**	**3.1%**	**0.40 [0.22, 0.73]**	
Total events	16		34				
Heterogeneity: Chi² = 0.86, df = 2 (P = 0.65); I² = 0%							
Test for overall effect: Z = 3.02 (P = 0.003)							
**3.13.4 Pulmonary Complications**							
feng 2016	0	11	1	8	0.1%	0.25 [0.01, 5.45]	2016
Ferrer 2016	0	65	8	65	0.7%	0.06 [0.00, 1.00]	2016
Bertoglio 2018	1	18	5	18	0.4%	0.20 [0.03, 1.55]	2018
Locham 2018	49	481	86	398	8.2%	0.47 [0.34, 0.65]	2018
Arnaoutakis 2019	5	92	16	66	1.6%	0.22 [0.09, 0.58]	2019
**Subtotal (95% CI)**		**667**		**555**	**11.1%**	**0.39 [0.29, 0.53]**	
Total events	55		116				
Heterogeneity: Chi² = 4.75, df = 4 (P = 0.31); I² = 16%							
Test for overall effect: Z = 6.12 (P < 0.00001)							
**3.13.5 Renal Complications**						Modified by random-effect model	
Ferrer 2016	6	65	8	65	4.4%	0.75 [0.28, 2.04]	2016
feng 2016	0	11	2	8	0.9%	0.15 [0.01, 2.76]	2016
Bertoglio 2018	1	18	2	18	1.4%	0.50 [0.05, 5.04]	2018
Locham 2018	65	481	138	398	8.2%	0.39 [0.30, 0.51]	2018
Geisbüsch 2018	418	839	827	1422	8.7%	0.86 [0.79, 0.93]	2018
Arnaoutakis 2019	6	92	24	66	5.2%	0.18 [0.08, 0.41]	2019
Kang 2019	1	68	4	54	1.5%	0.20 [0.02, 1.72]	2019
**Subtotal (95% CI)**		**1574**		**2031**	**30.3%**	**0.44 [0.24, 0.80]**	
Total events	497		1005				
Heterogeneity: Tau² = 0.35; Chi² = 49.26, df = 6 (P < 0.00001); I² = 88%							
Test for overall effect: Z = 2.70 (P = 0.007)							
**Total (95% CI)**		**6640**		**8398**	**100.0%**	**0.68 [0.63, 0.73]**	
Total events	679		1337				
Heterogeneity: Chi² = 109.29, df = 24 (P < 0.00001); I² = 78%							
Test for overall effect: Z = 10.51 (P < 0.00001)							
Test for subgroup differences: Chi² = 35.37, df = 4 (P < 0.00001), I² = 88.7%							

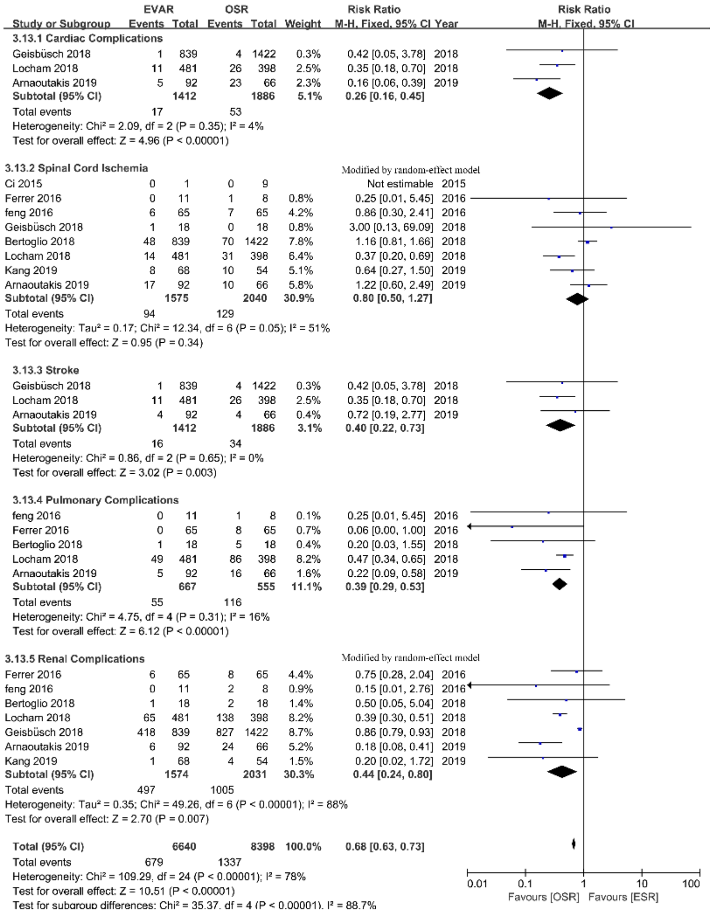

Figure S16 Forest plot comparing comparative complication rate during OSR, EVAR. The pooled risk ratios with 95% confidence intervals (CIs) were calculated using fixed-effects models and random-effects models. (OSR = Open Surgical Repair; EVAR = Endovascular Repair.)

Study or Subgroup	EVAR Events	EVAR Total	HSR Events	HSR Total	Weight	Risk Ratio M-H, Random, 95% CI	Year
**2.13.1 Cardiac Complications**							
Geisbüsch 2018	5	92	5	40	9.6%	0.43 [0.13, 1.42]	2018
Arnaoutakis 2019	26	839	7	346	10.3%	1.53 [0.67, 3.50]	2019
**Subtotal (95% CI)**		**931**		**386**	**19.9%**	**0.88 [0.26, 3.01]**	
Total events	31		12				
Heterogeneity: Tau² = 0.53; Chi² = 2.96, df = 1 (P = 0.09); I² = 66%							
Test for overall effect: Z = 0.21 (P = 0.84)							
**2.13.2 Spinal Cord Ischemia**							
Geisbüsch 2018	8	11	1	24	7.8%	17.45 [2.48, 122.99]	2018
Arnaoutakis 2019	48	68	17	346	10.8%	14.37 [8.82, 23.41]	2019
Kang 2019	30	92	4	40	10.0%	3.26 [1.23, 8.65]	2019
**Subtotal (95% CI)**		**171**		**410**	**28.6%**	**8.80 [2.76, 28.03]**	
Total events	86		22				
Heterogeneity: Tau² = 0.73; Chi² = 7.90, df = 2 (P = 0.02); I² = 75%							
Test for overall effect: Z = 3.68 (P = 0.0002)							
**2.13.3 Stroke**							
Arnaoutakis 2019	4	92	1	40	7.4%	1.74 [0.20, 15.07]	2019
**Subtotal (95% CI)**		**92**		**40**	**7.4%**	**1.74 [0.20, 15.07]**	
Total events	4		1				
Heterogeneity: Not applicable							
Test for overall effect: Z = 0.50 (P = 0.62)							
**2.13.4 Pulmonary Complications**							
Arnaoutakis 2019	5	92	11	40	10.0%	0.20 [0.07, 0.53]	2019
**Subtotal (95% CI)**		**92**		**40**	**10.0%**	**0.20 [0.07, 0.53]**	
Total events	5		11				
Heterogeneity: Not applicable							
Test for overall effect: Z = 3.21 (P = 0.001)							
**2.13.5 Renal Complications**							
feng 2016	418	839	245	346	11.0%	0.70 [0.64, 0.77]	2016
Geisbüsch 2018	1	68	2	24	6.9%	0.18 [0.02, 1.86]	2018
Arnaoutakis 2019	0	11	2	3	6.0%	0.07 [0.00, 1.12]	2019
Kang 2019	6	92	16	40	10.2%	0.16 [0.07, 0.39]	2019
**Subtotal (95% CI)**		**1010**		**413**	**34.1%**	**0.26 [0.08, 0.88]**	
Total events	425		265				
Heterogeneity: Tau² = 1.00; Chi² = 15.52, df = 3 (P = 0.001); I² = 81%							
Test for overall effect: Z = 2.16 (P = 0.03)							
**Total (95% CI)**		**2296**		**1289**	**100.0%**	**0.97 [0.35, 2.67]**	
Total events	551		311				
Heterogeneity: Tau² = 2.44; Chi² = 198.02, df = 10 (P < 0.00001); I² = 95%							
Test for overall effect: Z = 0.07 (P = 0.95)							
Test for subgroup differences: Chi² = 27.87, df = 4 (P < 0.0001), I² = 85.6%							

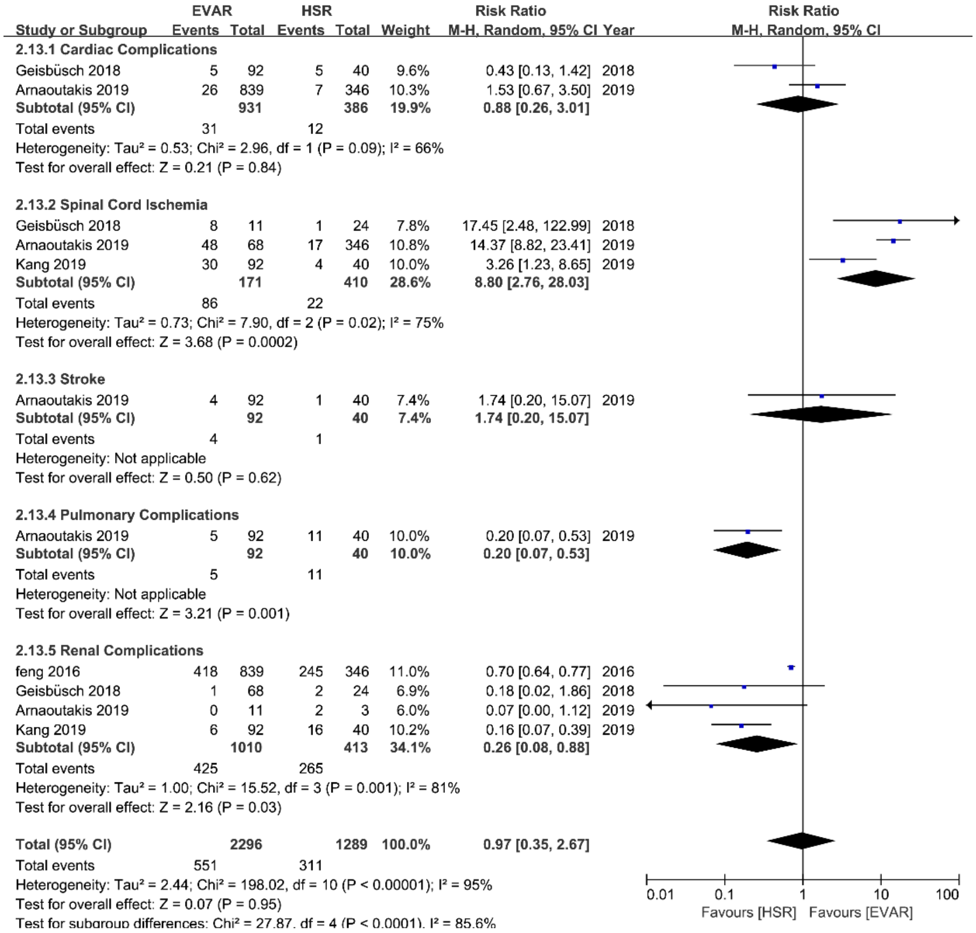

Figure S17 Forest plot comparing comparative complication rate during HSR, EVAR. The pooled risk ratios with 95% confidence intervals (CIs) were calculated using fixed-effects models and random-effects models. (HSR = Hybrid Surgery Repair; EVAR = Endovascular Repair.)

Table S4 – Final GRADE Assessment for perioperative mortality comparison between the 3 treatment options

Each comparison in the network is graded in turn on a scale of “Very low” -> “Low” -> “Moderate” -> “High” certainty. As per the GRADE guidelines, the starting rating for all comparisons is “Low” due to the non-randomised nature of the included studies:

1. EVAR vs OPEN

A) <u>Rating the direct estimate *(RR 0.44 95%CI 0.36-0.53):* Moderate</u>

Downgrading factors:
Risk of bias: some concern
Heterogeneity: no concern
Indirectness: no concern
Publication bias: no concern

Upgrading factors:
Obvious confounding would relate to physiological risk: open surgery is generally performed in fitter patients. Therefore, to adjust for this would only increase the effect noted (that open surgery has worse perioperative mortality than EVAR). This therefore upgrades the rating one position for this outcome measure.

B) <u>Rating the indirect estimate: Very Low</u>

The most dominant first order loop is EVAR -> HSR -> OPEN:
EVAR vs HSR rating (direct): Very Low
OPEN vs HSR rating (direct): Very Low

Transitivity: low concern

C) <u>Rating the network estimate *(RR 0.37 95%CI 0.22-0.71)*: Moderate</u>

Highest between direct/indirect ratings: Moderate
Incoherence: No concerns
Imprecision: No concerns

2. HSR vs OPEN

A) Rating the direct estimate *(RR 1.23 95%CI 1.04-1.45)*: Moderate

Downgrading factors:
Risk of bias – some concern
Heterogeneity – no concern
Indirectness – no concern
Publication bias – no concern

Upgrading factors:
Obvious confounding would relate to physiological risk: open surgery is generally performed in fitter patients. Therefore, to adjust for this would only increase the effect noted (that open surgery has better perioperative mortality than HSR). This therefore upgrades the rating one position for this outcome measure.

B) Rating the indirect estimate: Very Low

The most dominant first order loop is HSR -> EVAR -> Open:
Open vs EVAR rating (direct): Very Low
EVAR vs HSR rating (direct): Very Low

Transitivity: low concern

C) Rating the network estimate: *(RR 1.22 95%CI 0.66-1.98)*: Low

Highest between direct/indirect ratings: Moderate
Incoherence: No concerns
Imprecision: Major concerns

3. EVAR vs HSR

A) **<u>Rating the direct estimate *(RR 0.34 95%CI 0.26-0.43)*: Low</u>**
Downgrading factors:
Risk of bias – some concern
Heterogeneity – no concern
Indirectness – no concern
Publication bias – no concern

B) **<u>Rating the indirect estimate: Very Low</u>**
The most dominant first order loop is EVAR -> OPEN -> HSR:
EVAR vs OPEN rating (direct): Very Low

OPEN vs HSR rating (direct): Very Low

Transitivity: low concern

C) **<u>Rating the network estimate *(RR 0.31 95%CI 0.22-0.71)*: Low</u>**
Highest between direct/indirect ratings: Low
Incoherence: No concerns
Imprecision: No concerns
